# Development and selective grain make plasticity 'take the lead' in adaptive evolution

**DOI:** 10.1186/s12862-021-01936-0

**Published:** 2021-11-20

**Authors:** Miguel Brun-Usan, Alfredo Rago, Christoph Thies, Tobias Uller, Richard A. Watson

**Affiliations:** 1 Institute for Life Sciences/Electronics and Computer Sciences, University of Southampton, Southampton, UK; 2 Department of Biology, Lund University, 22362 Lund, Sweden

**Keywords:** Adaptation, Phenotypic plasticity, Genotype-phenotype-map, Plasticity-led evolution, Parental effects, Mechanistic developmental models

## Abstract

**Background:**

Biological evolution exhibits an extraordinary capability to adapt organisms to their environments. The explanation for this often takes for granted that random genetic variation produces at least some beneficial phenotypic variation in which natural selection can act. Such genetic evolvability could itself be a product of evolution, but it is widely acknowledged that the immediate selective gains of evolvability are small on short timescales. So how do biological systems come to exhibit such extraordinary capacity to evolve? One suggestion is that adaptive phenotypic plasticity makes genetic evolution find adaptations faster. However, the need to explain the origin of adaptive plasticity puts genetic evolution back in the driving seat, and genetic evolvability remains unexplained.

**Results:**

To better understand the interaction between plasticity and genetic evolvability, we simulate the evolution of phenotypes produced by gene-regulation network-based models of development. First, we show that the phenotypic variation resulting from genetic and environmental perturbation are highly concordant. This is because phenotypic variation, regardless of its cause, occurs within the relatively specific space of possibilities allowed by development. Second, we show that selection for genetic evolvability results in the evolution of adaptive plasticity and vice versa. This linkage is essentially symmetric but, unlike genetic evolvability, the selective gains of plasticity are often substantial on short, including within-lifetime, timescales. Accordingly, we show that selection for phenotypic plasticity can be effective in promoting the evolution of high genetic evolvability.

**Conclusions:**

Without overlooking the fact that adaptive plasticity is itself a product of genetic evolution, we show how past selection for plasticity can exercise a disproportionate effect on genetic evolvability and, in turn, influence the course of adaptive evolution.

**Supplementary Information:**

The online version contains supplementary material available at 10.1186/s12862-021-01936-0.

## Background

Understanding how evolution works is not complete by understanding natural selection; we also need to understand the generation of the phenotypic variation that natural selection will act on [1, 2]. While the genetic variation that makes evolution possible can be considered non-directional (i.e. “random mutations”), the phenotypic variation that results from these genetic changes is highly structured, causing some variants to appear more frequently than others [3–5]. This directional phenotypic variation arises because of the complex process of development, and is hence commonly known as “developmental bias” [6, 7]. If this developmental bias is aligned with the adaptive demands imposed by local environments (i.e., if the mutationally more accessible phenotypes are also the more adaptive ones), adaptive evolution would be greatly facilitated. Here, we refer to this as a situation with high genetic evolvability, whereas a situation where adaptive phenotypes would be total or partially inaccessible through mutation represent low genetic evolvability.

The developmental origins of genetic evolvability, and the causes and consequence of its evolution, have generated much interest and controversy, not the least over the past decade [6, 7]. One reason for the controversy is that it is unclear why developmental systems should exhibit facilitated variation. Genetic evolvability could itself be a product of past evolution [7–9], but the idea that natural selection would be able to improve genetic evolvability is problematic because the immediate selective gains of responding adaptively to random genetic change are small on short timescales [9, 10].

Another suggestion is that the high genetic evolvability is acquired through the capability of organisms to rapidly adjust to their environment during their lifetime [11]. However, while adaptive phenotypic plasticity often appears to ‘take the lead’ in adaptive evolution (e.g., [12, 13]), the idea that adaptive plasticity explains genetic evolvability overlooks the need to explain the origination of the adaptively plastic response that supposedly ‘came first’. In this paper, we seek to better understand whether phenotypic plasticity can help to explain genetic evolvability without overlooking the fact that adaptive plasticity is itself a product of genetic evolution [14].

The starting point of our approach is grounded on previous theoretical and empirical observations which suggest that the phenotypic consequences of genetic variation and environmental variation are not independent [12, 14–18]. Such non-independence is indeed expected, because the consequences of any perturbation of development will be channelled by the same underlying developmental mechanisms [19]. From this, it follows that, if selection for phenotypic plasticity alters the structure of developmental interactions, this will likely alter how phenotypes respond to genetic mutations and hence affect evolvability. Population genetic models demonstrate that selecting for plasticity can increase genetic variation along the dimensions of the phenotype that are plastic [20, 21]. This seems to support a role for plasticity in shaping genetic evolvability. However, given that the interdependence of plasticity and evolvability on development is essentially symmetric (i.e. both covary as a result of a common developmental dynamics, but none is cause or consequence of the other), the reverse may be equally possible. That is, if selection on genetic evolvability alters the structure of developmental interactions, this should also alter how phenotypes respond to environmental variation. It thus remains an open question whether genetic evolvability is predominantly shaped by plasticity or vice versa [14, 22].

To address this question, we study the relationship between plasticity and evolvability by representing these phenomena in a common framework where the phenotypic effects and adaptive consequences of genetic and environmental variation can be compared. The phenotype distribution that is generated by genetic variation can be represented as a genotype-phenotype (GP) map: an idealized representation of development that assigns a phenotype to each genotype [3, 6, 22]. If the main axis of the phenotypic distribution showed by the GP map is aligned with the adaptive demands, then phenotypes that are suitable for adaptation arise more readily, and the GP map is said to exhibit high genetic evolvability [9].

Analogous to the GP map, plasticity can be understood as an Environment-Phenotype (EP) map (aka reaction norm) that associates each environment with its corresponding phenotype [23–25]. Here, a developmental system is considered “plastic” if it reacts to different environmental inputs producing different phenotypes (the more disparate those phenotypes the more “plastic” the system and the “steeper” its reaction norm in the trait-space). Non-plastic systems will always produce the same phenotype irrespective of the environment (i.e. single-point EP-maps). Plasticity is adaptive when the EP map enables individuals to produce phenotypes that fit the requirements of the environment in which they find themselves. For instance, if plasticity is adaptive, organisms receiving biophysical or biochemical cues from an environment A would develop a phenotype that is highly advantageous in that environment A, and the same for environments B, C, etc. As detailed below, those plastic responses can change during evolutionary timescales (i.e. they can be “evolvable”), so that non-adaptive forms of plasticity eventually become adaptive if the proper selective pressures are applied (but the rate of adaptation may vary with the system considered).

However, the GP and EP maps do not exhaust the sources of phenotypic variation. Development is also sensitive to non-genetic and non-environmental initial conditions such as biochemical templates, resources and nutrients that are provided by the parents [26, 27]. The association of such heritable epigenetic elements with phenotypic variation is often referred to as parental effects [28]. Here we emphasize the analogous role of such initial conditions in structuring the phenotype distribution by referring to it as the PP (for ‘Parental-Phenotype’) map: a mapping that associates a phenotype with the parentally inherited initial conditions required to produce it. Notice that, although “maps” are formal mathematical objects, we adopt here a more flexible use of the term, equating it to the phenotypic distributions arising from a specific type of parametric perturbations.

To model the potential interdependence of these three maps (GP, EP and PP), we use several different and widely used models of development based on gene regulatory networks (GRN). This approach means that we neither assume that the three maps are independent nor that they are related; rather, these are hypotheses we can test. To do this we apply the three different forms of variation (i.e. genetic, environmental, parental) to the core GRNs of the developmental system we use, and compare the resulting phenotypic effects to see if they are similar or not.

In principle, it could be the case that any concordance between the three maps in this model could be intrinsic to the properties of GRNs and arise without selection, or observed only in GRNs subjected to particular selective conditions, or not observed at all. Likewise, it is not known whether selection can, for example, change the GP map produced by a GRN without altering the EP or PP maps, or more generally, whether the effects of selecting for one map has consequences for the properties of the others. Lastly, even if it is the case that selection for one map can determine the evolution of any other map, it could be the case that one of the maps is easier to change with selection than the others. Therefore, the aims of this paper are (1) to assess the potential interdependence of the GP, EP and PP maps; (2) to determine how (and if) the evolution of one map affects the evolution of the others, and whether this evolution is symmetric; and (3) to establish whether or not the three maps are equally responsive to selection.

## Results

**Fig. 1:**
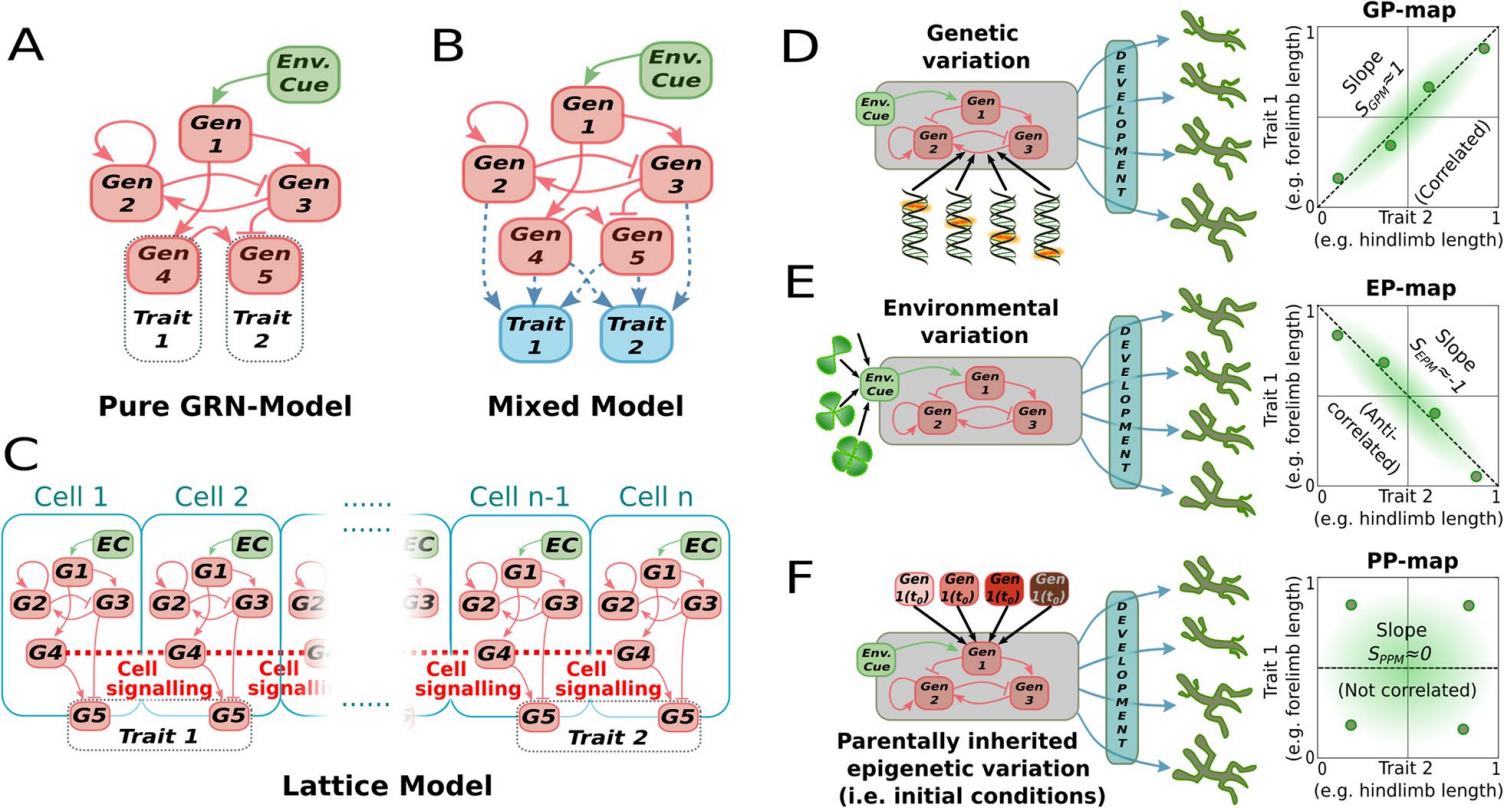
Experimental overview. Conceptual depiction of the three GRN-based models used in this work: **A** A pure GRN model where the (two-trait) phenotype is the steady-state concentration of two arbitrary genes. **B** GRN + Multilinear model, where each phenotypic trait is calculated as the weighted sum of all the elements within the steady-state GRN. **C** Lattice model, where the phenotype is conceptualised as the steady-state expression pattern of one of the constituent genes (Gen 5 in this example) along a one-dimensional row of cells that can communicate between them through cell–cell signalling. In all of these models, phenotypic variation is created by perturbing one or several elements in the core GRN: Perturbations can be introduced in the strength of gene–gene interaction (i.e. as genetic mutations, **D**); in some environmental cue that may regulate some environmentally-sensitive gene (**E**); or in the (maternally inherited) initial concentrations of each GRN element (**F**). Perturbations on each of these three different sources of phenotypic variation (one element of the GRN perturbed at a time) will produce a collection of two-trait phenotypes (i.e., hind- and fore-limb lengths). If these phenotypes are plotted in a two-trait (*T*_*1*_*-T*_*2*_) morphospace, they can reveal the structure of the parameter-to-phenotype maps (**D**–**F**, right panels). The linear slopes of these maps can be used as a coarse description of these maps, allowing for map-to-map comparisons of random (Fig. 2) and evolved GRNs (Figs. 3, 4, 5)

To model how the GP, EP and PP maps interact, it is necessary to represent developmental systems in a way that allow for genetic, environmental and parental inputs. We expand on three different and widely used GRN-based models of development, each one entailing a different complexity and biological realism (Fig. 1A–C). To better interpret our results, we succinctly introduce here the architecture of the models we use (see “Methods” section for further details). From the simpler to the more complex, these models are:

### Basic GRN model

This model (based on [29]) represents a simple gene regulatory network (GRN; Fig. 1A). It consists of *N*_*g*_ transcription factors that have continuous, positive concentrations (vector *G* = *(g*_1_*,…,g*_*Ng*_*); gi* ≥ *0 ∀ i*), and regulate the expression of each other by binding to cis-regulatory sequences on gene promoters. The regulatory interactions of this GRN are encoded in the *N*_*g*_* x N*_*g*_ matrix *B*, whose elements *B*_*ik*_ represent the effect of gene *k* on the transcription of gene *i*. Positive elements (*B*_*ik*_ > *0*) represent activation and negative elements (*B*_*ik*_ < *0*) represent inhibition. A binary (0 or 1) matrix *M* (*N*_*g*_* x N*_*g*_) encodes the GRN topology, so that the interaction *B*_*ik*_ is only active if *M*_*ik*_ = *1*. The initial state of the vector *G* (represented by the vector *G*_*0*_) accounts for the initial state at the beginning of development, which is supposed to be parentally determined. In addition, the expression of each gene *j* can be potentially modulated by an environmental factor *E*_*j*_, which can either upregulate (0 ≤ *E*_*j*_ ≤ *1*) or downregulate it (*−1* ≤ *E*_*i*_ ≤ *0*). The environmental effects of all these *N*_*e*_ environmental factors *(N*_*e*_ = *N*_*g*_*),* are contained within the vector *E.*

Developmental dynamics are attained by changes in gene concentration over a number of developmental iterations (*t*_*dev*_), and the phenotype is recorded as the steady-state expression levels of two arbitrarily chosen genes in *t*_*dev*_ (Fig. 1A). Only viable (temporally stable) phenotypes are considered: from 1 to *N*_*g*_, the normalized *G* (represented here as G*), must remain the same within a threshold of *10*^*–2*^ over an interval of *t*_*dev*_/10 developmental time units (|G*_0.9·tdev_-G*_tdev_*|*≤ *10*^*–2*^). The gene–gene interactions within the GRN follow a non-linear, saturating Michaelis–Menten dynamics (a special type of Hill function), so that the concentration of the gene *i* changes over developmental time according to the following differential equation:

1 $$\frac{{\partial g_{i} }}{\partial t} = \frac{{R\left( {h_{i} } \right)}}{{K_{M} + R\left( {h_{i} } \right)}} - \mu g_{i} + \xi$$

where

2 $$h_{i} = \sum\nolimits_{j = 1}^{Ng} {M_{ij} B_{ij} g_{j} + E_{j} }$$

and *R (h*_*i*_*)* is the Ramp function (*R(x)* = *x, ∀ x* ≥ *0* and 0 otherwise) which prevents negative concentrations in gene products resulting from inhibiting genetic or environmental interactions. *K*_*M*_ is the Michaelis–Menten coefficient. Without loss of generality, we set *K*_*M*_ = 1 (other choices of *K*_*M*_ or specific Hill functions are known not to affect the results, see [30]). The environmental term *E*_*j*_ is embedded within the gene-ordered summation of Eq. (2) following the associative property of the sum, and because *N*_*e*_ ≤ *N*_*g*_ always. Notice that, while environmental factors can effectively inhibit gene expression (if *E*_*j*_ < *0*), they cannot turn a genetic concentration into a negative value (because of the Ramp function R). All genes and gene products (but not environmental factors) are degraded with a decay term *μ* = *0.1.* In order to avoid unstable solutions, a certain amount of (Gaussian) noise is introduced in the system through the term *ξ,* randomly drawn from a Normal distribution ~ *N(0, 10*^*–2*^*).*

### GRN + multilinear model

This mixed model (based on [21]), can be viewed as a multi-linear model of phenotypic determination [20] that is added to a basic GRN-model [29] (Fig. 1B). The key difference with the previous model lies in how each phenotypic trait is generated. Rather than being the expression level of one element of the GRN, each trait *T*_*i*_, *i* = (*1*, *2*) receives a contribution from each transcription factor according to a linear coefficient:

3 $$T_{i} = \sum\nolimits_{j = 1}^{Ng} {} Z_{ij} g_{j}$$

where the factor *Z*_*ij*_ represents the contribution of the *jth* gene to the *ith* trait (*−1* < *Z*_*ij*_ < *1*). Note that the *Z* matrix encoding the linear coefficients is separated from the matrix *B* encoding the GRN itself. In this paper, the evolutionary implications of the correlations between maps are reported on the basis of this model.

### Lattice model

This reaction–diffusion model (based on [30, 31]) represents a simple developmental model that implements multicellular phenotypes in an explicitly spatial context (Fig. 1C). The model describes on a one-dimensional row of *N*_*c*_ non-motile cells (*N*_*c*_ = 16 in our case), whose developmental dynamics is determined by a GRN (as described in the basic model) that is identical for all cells. Interaction between the different cells is achieved through cell–cell signalling involving extracellular diffusion of morphogens (*N*_*g*_*/3* of the GRN elements are considered to be diffusible morphogens). Each of these morphogens has a specific diffusion rate *D*_*i*_ (*0* < *D*_*i*_ < *1*) and follows Fick’s second law. Zero-flux boundary conditions are used. Thus, the concentration of gene *i* over developmental time now is calculated as:

4 $$\frac{{\partial g_{ij} }}{\partial t} = \frac{{R\left( {h_{ij} } \right)}}{{K_{M} + R\left( {h_{ij} } \right)}} - \mu g_{ij} + \xi + D_{i} \nabla^{2} g_{ij}$$

In most works that use this model, the phenotype is conceptualised as the expression pattern of one of the constituent genes along the row of cells (e.g. [30, 31]). Here, for the sake of comparability with the other models used, we set *T*_*1*_ and *T*_*2*_ as the average concentration of gene 1 in the first and last two cells of the organism (*T*_*1*_ = *(g*_*1,1*_ + *g*_*1,2)*_*/2*, and *T*_*2*_ = *(g*_*1*_*,*_*Nc-1*_ + *g*_*1*_*,*_*Nc*_*)/2)*.

While these models differ in complexity, all three feature a GRN at their core, which is defined by three types of variables—namely, regulatory connections, initial gene expression and exogenous inputs (see “Methods” and Fig. 1). Together, these variables define the GRN dynamics which, when implemented and iterated in the models, result in measurable phenotypes. Furthermore, these three types of variables have some correspondence with the three types of variation we address (genetic, parental and environmental). For example, parental effects generally apply modification only in early stages, and thus can be allied to the initial gene expression values of the GRN. This provides an intuitive way to link each of the constituent elements of the GRN to a different source of phenotypic variation: (i) changes in gene–gene interaction strengths in the GRN can be conceptualised as an effect of genetic variation; (ii) changes in the environmentally sensitive elements in the GRN (sensor nodes and diffusion rates) as an effect of environmental variation; and (iii) changes in the initial concentration of each transcription factor in the GRN as an inherited initial state (Fig. 1D–F).

However, it is also true that, for example, an environmental input might change a regulatory sensitivity and a genetic mutation might change an initial gene expression level, rendering the correspondence non-univocal. This loose correspondence should be kept in mind in interpreting our results, but we will show that the type of model variable itself entails a marginal explanatory power when compared with other factors (i.e. the timescale of the change in these variables, see next sections). In addition, even when the correspondence is not strictly one-to-one, our approach exhausts the ways in which a specific GRN can vary (these variations do not alter the GRN topology, which here is assumed to evolve much slower than the inputs [4, 8]).

While more complex models (e.g. cell-based models including morphogenesis) could implement environmental or parental inputs in the developmental dynamics in more ways than we consider here (e.g. by changing the bio-physical properties of cells and tissues), the size of their parameter spaces and their associate computational costs would render our approach unfeasible. Notwithstanding this limitation of our work, comparing the results for these three different models allows us to assess how the robustness of our results escalates with model complexity (see Additional file 1: Fig. S2).

With the described settings, all the models used in this paper produce a single, 2-trait (2-dimensional) phenotype for each combination of inputs. Thus, a set of phenotypes (i.e. a phenotype distribution) can be generated by introducing variation in those inputs. These phenotype distributions represented in a 2D morphospace are considered maps: those resulting from variation in the genetic inputs are GP maps, whereas those resulting from environmental perturbations or perturbations in the initial conditions are considered as EP and PP maps, respectively. While for some authors (e.g. [31, 32]) any map exhibiting phenotypic variation in response to genetic mutations has the property of being evolvable; we consider a GP map to exhibit more or less *genetic evolvability* to the extent that its phenotypic distributions are aligned with the adaptive demands (in this sense, evolvability is a joint property of variation + selective environment, not a property of the map alone; [9, 10]). If a similar alignment is found in the EP map or the PP map, they are said to exhibit adaptive plasticity and adaptive parental effects, respectively.

Considered together, the three maps encompass the whole set of phenotypes that a given developmental mechanism can generate from the sum of all perturbations. This “total” variation, which can be also represented in a 2D trait space, is referred to as a general phenotype distribution, or GPD (i.e., the GP, EP and PP maps are all contained within this GPD, Additional file 1: Fig. S1).

### GP, EP and PP mappings are correlated in randomly generated GRNs

We first explore the inter-dependence between GP, EP, and PP maps in a large (*n* > *10*^6^) ensemble of randomly generated GRNs (encompassing different GRN connectivities, topologies and number of genes, see “Methods”). We then separately introduced random variation (10 input values *0* < *x* < *1*) in the genetic, environmental and parental inputs (i.e., connection weights, expression levels, and initial conditions, respectively) of each of these GRNs, and compared the resulting phenotypic distributions, that is, the resulting GP, EP and PP maps.

**Fig. 2:**
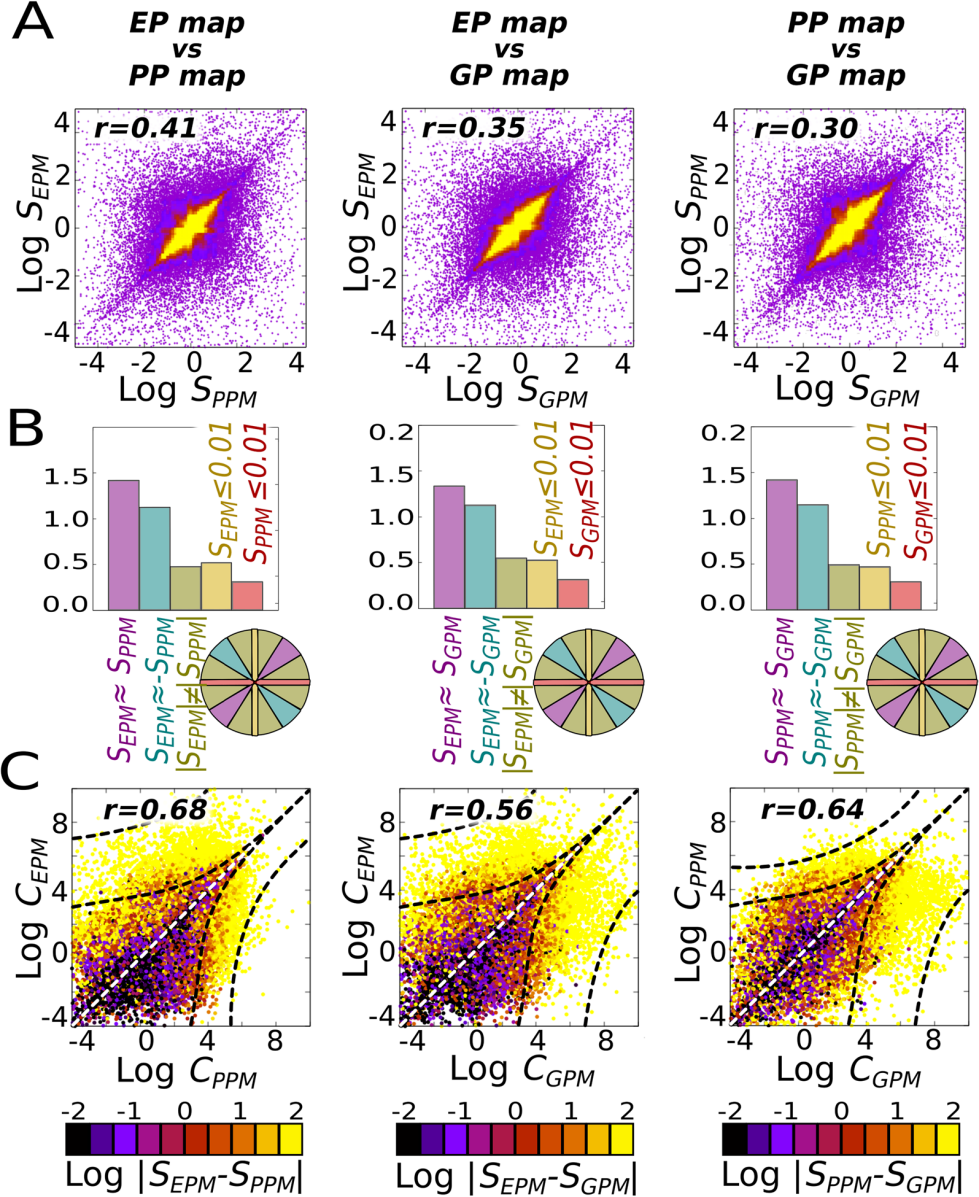
Phenotypic distributions arising from genetic, epigenetic or environmental perturbations are not independent. In a large random ensemble of GRNs (*n* = *10*^*6*^), systematic parametric variations were introduced into each of their elements. Each perturbation on an element generates a collection of phenotypes in a two-trait morphospace (a *XPM* map), characterised by a linear slope *S*_*XPM*_ (see Fig. 1D–F). **A** For each GRN, we compare these slopes, two by two, searching for their correlations in the two-slope spaces (note that these are not two-trait morphospaces). Each dot is a GRN, and the yellow shaded region contains 90% of the GRNs. Correlations are significant (Pearson *r* > 0.3) for every combination of maps considered. **B** Histograms showing the probability distribution of maps with developmental insensitivity to the first (*Sx* ≤ 0.01; yellow) or second (*Sy* ≤ 0.01; red) type of inputs; and of correlated (parallel slopes); anti-correlated (perpendicular slopes) and non-correlated slopes (otherwise). Each of these cases correspond to the sector of a hypothetical circumference engulfing all points of (**A**), as exemplified in the coloured circumference, and the relative frequency represents the probability of each point to be located within each sector. **C** The complexities of the parameter-to-phenotype maps (i.e., how non-linear they are, see “Methods”); rather than between their linear slopes are also positive (Pearson *r* > *0.56*). In (**C**), the colour represents slope similarity: similar slopes (black colour) are associated to simpler (i.e., more linear) maps. *n* = *30* replicates, GRN + multilinear model (see Additional file 1: Fig S2 for correlations under other models and Additional file 1: Fig. S5 for a null model on **C**)

We estimated the similarity between these three maps by testing whether or not variation in the genetic, environmental or parental inputs produce similar covariation between the two traits (Fig. 1D–F), using the linear slopes in the phenotypic morphospace as basic descriptors of the different maps. Since comparisons were performed using the (linear, unordered) map slopes, and not the whole phenoypic distributions of each map, we used a simple (Pearson) product moment correlation to evaluate whether or not the slope of a map was associated with a similar slope in the other maps. Pairwise comparisons between the slopes caused by variation in genetic, environmental, or parental inputs were all significantly positive (Pearson *r* ≥ 0.3; Fig. 2A). This demonstrates that GP, EP and PP mappings are not independent in random GRNs. Note that this positive correlation between maps does not imply that the map-specific slopes themselves are positive; only that their slopes, which can be positive or negative, are similar (a comparison between maps that does not consider the direction of the slope since genetic evolvability is concerned with the sensitivity to random mutations, not their direction; [9]). GRNs showing zero or negative correlation between different mappings also exist, but they are less frequent (Fig. 2B). Importantly, this interdependence across different mappings is robust to more detailed measures of map-to-map similarity, such as Euclidean distances (ED, Additional file 1: Fig. S3). Such ED-based correlations, however, decrease as the difference between the map slopes become too large (presumably as an effect of map complexity itself, since complex maps are more dissimilar than simpler ones, see next section, Fig. 2C, Additional file 1: Fig. S3).

To eliminate the possibility that these observed correlations were caused by similarities in the input values, rather than in the structure of the GRNs, we gradually randomized the input parametric values while recording the correlations between maps (Additional file 1: Fig. S4). This procedure revealed that the correlations do not depend on particular choices of the input parameters. In contrast, correlations were extremely sensitive to parametric changes in the GRN topology, suggesting that the observed similarity between maps is caused by the structure of the GRN connections (i.e., ‘developmental mechanism’ sensu [30–32]) rather than the structure of the input perturbations. While a relationship between specific GRN topologies and the strength of the map-to-map correlations is expected (as it occurs for individual maps [30, 31]), further studies would be required to establish this relationship in more detail.

Another alternative explanation for our results would be that the observed correlations are driven by the (close-to-zero) linear slopes associated with very complex (i.e. a “zigzag”-like) maps. However, Fig. 1A shows that even the central region of the correlational space where most (> 90%) maps are contained shows a clear diagonal structure densely populated with non-zero (*1* <*|S*_*x*_*|*< *2*) slopes. Furthermore, if this would be the case, one would expect Euclidean distances between maps to be generally very large (i.e. because most of them would be very complex and highly dissimilar maps having both close-to-zero slopes). This possibility is rejected by our observations (Additional file 1: Fig S3B), which show that the majority of map-to-map Euclidean distances occurs at quite low values (*ED*_*A,B*_*≈2*), and is mostly constituted by relatively simple, sub-linear maps).

Finally, our simulations reveal that map-to-map correlations are affected in non-trivial ways by certain GRN features, such as GRN size, connectivity, the number of iterations in the GRN dynamics or the model architecture (Additional file 1: Fig. S2). In general, correlations decrease as connectivity and GRN size increase (presumably because large networks offer more opportunities for modularity, which in turn may enable a developmental de-coupling between different traits).

### The complexity of GP, EP and PP maps are correlated in randomly generated GRNs

We also investigated whether or not the GP, EP and PP maps exhibit similar complexity in random GRNs. We defined map complexity as the degree of non-linearity in phenotypic response to inputs. This captures the intuition that a linear slope is less complex than a U-shaped response, which is itself simpler than a W-shaped response. Comparing the map complexities between the 10^6^ random GRNs reveals that map complexities are, on average, positively correlated (Pearson *r* ≥ 0.56; Fig. 2C and Additional file 1: Fig. S2). In other words, if a map (e.g., GP) is simple, the other maps (EP and PP) will be simple too, and they will exhibit very similar slopes. In contrast, if a map is complex, other maps too are likely to be complex, and their slopes will be less similar (Fig. 2C and Additional file 1: Fig. S2).

To ensure that these observed correlations between map complexities are not a general property of pairs of input–output maps, we analysed a large ensemble of random mathematical functions (polynomials of known degree ≤ 4) using the same tools that we used for calculating map complexity. This analysis verified that the correlations do not arise between pairs of randomly selected functions unless they belong to the same complexity class (polynomial degree) (Additional file 1: Fig. S5).

How a network topology creates similarity between map slopes and complexities can be better understood by looking at the whole set of developmentally attainable phenotypes (general phenotypic distribution: GPD), which can be revealed by means of massive and unspecific parametric perturbations (see “Methods” and Additional file 1: Fig. S1). This procedure shows that each generative network creates a distinctive GPD with a highly anisotropic and discontinuous structure. This structure increases the likelihood that individual maps will have similar slopes simply because many phenotypic directions of change are either very unlikely or developmentally impossible (Additional file 1: Figs. S1, S2, S4 and S5).

Positive map-to-map correlations in both slopes and map complexities were found in all three considered models of phenotypic determination (Additional file 1: Fig. S2). However, the correlation coefficients are higher and more variable for complex models involving more than pure-GRN dynamics (Figs. 1B–C and Additional file 1: Fig. S2).

### Evolving only one of the GP, EP or PP maps changes the phenotypic biases across the other maps

After exploring the map-to-map correlations in random GRNs, we next wanted to address whether or not adaptive changes within one map (i.e., changes in the covariation between traits) are able to induce similar changes to the other maps. To do so, we performed three sets of selection simulations using the developmental model of intermediate complexity (GRN + multilinear). This model was chosen because it is the one showing more stable map-to-map correlations under a wider range of assumptions and GRN properties (see Additional file 1: Fig. S2). In each simulation, we allowed only one of the three different maps (henceforth the “selected map”) to evolve in response to selection. We refer to the other maps as the “non-selected” maps (see “Methods” for details).

Although an individual may experience many environmental inputs during its lifetime, it has only one genotype and, generally, one parental input (here initial condition). This means that, in one generation, natural selection can act on a distribution of environmentally induced phenotypes, but only on a single phenotype produced by genetic variation (that is why the evolution of GP and PP maps would ordinarily require lineage selection over many generations). Our main point in this paper depends, indeed, on the fact that this difference in the selective timescale makes selection for phenotypic plasticity likely to be a strong driver of genetic evolvability, but not vice versa (see next sections). But, before we get to that, it is necessary to first examine how evolving one map influences other maps, and to examine this properly it is necessary to be able to apply an equally effective, fine-grained selection on each of the selected maps. While introducing similar rates of change in the genetic, environmental and parental inputs is biologically unrealistic, it enables us to examine the evolutionary interdependence of the maps that arise because of their developmental linkage, while removing the differences in their capacity to be selected (next experiments will address more biologically grounded cases).

**Fig. 3:**
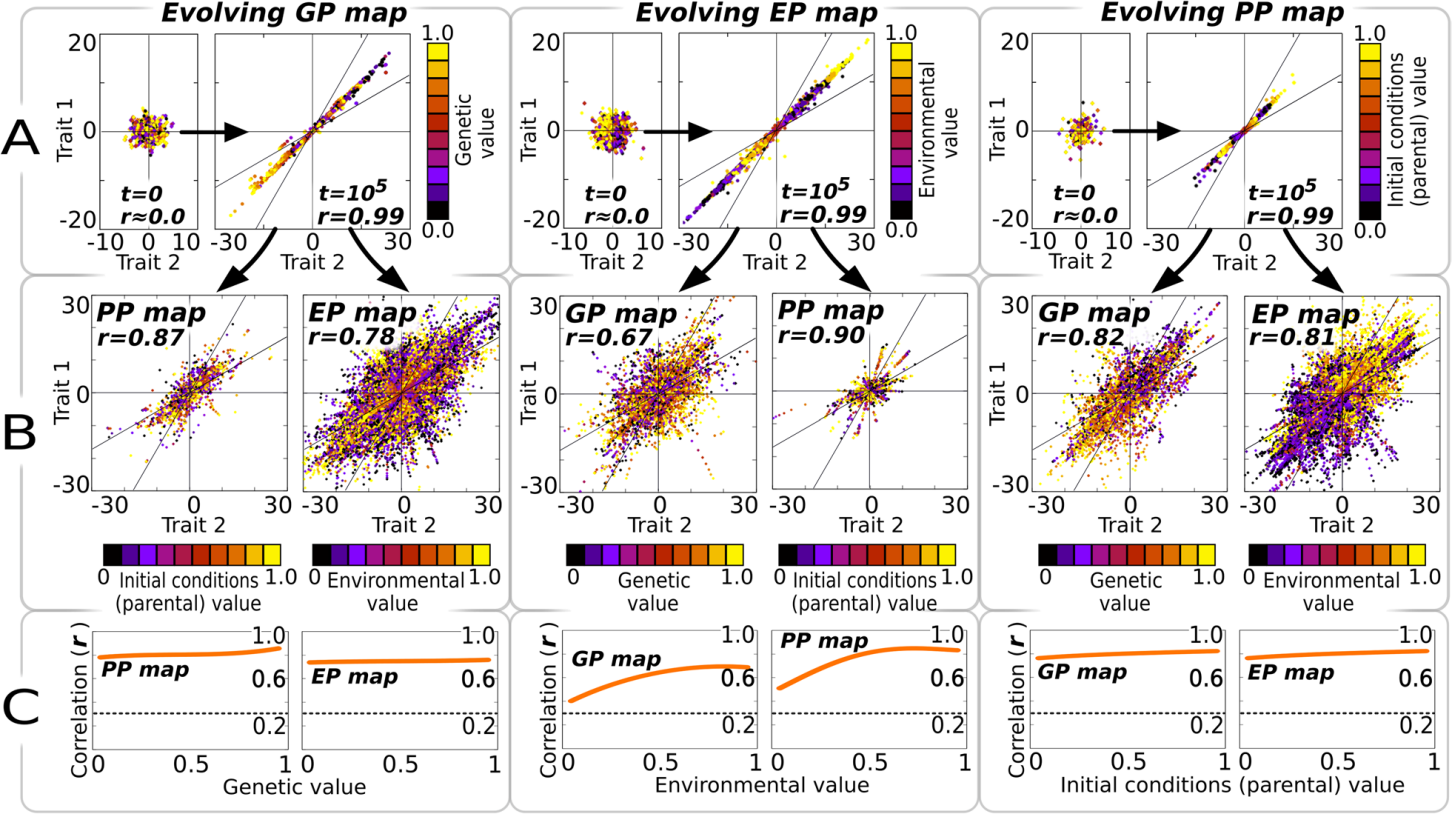
Evolving a single map creates similar phenotypic distributions in the other maps. A population whose individuals initially exhibit a random phenotypic distribution in *t* = *0* (**A**, small panels) is evolved to fit a target phenotypic distribution (*S*^*T*^ = *1*) using as an input just one kind of phenotypic determinant (i.e., genetic, environmental, or parental variation). Other targets (*S*^*T*^ = *−1*) give similar results (see Additional file 1: Fig. S6). In each generation, one individual is exposed to 10 different input values (*0* < *x* < *1*) of a single phenotypic determinant (the colour of each dot in **A**–**B** represents value of this input). This parametric variation produces a set of ten potential phenotypes whose slope is compared to the target to evaluate the individual’s fitness (see “Methods”). After *10*^*5*^ generations in a mutation-selection-drift scenario (where other sources of phenotypic variation are frozen), the population has a narrow phenotypic distribution in the evolved map (**A**, large panels). In (**B**) we uncover variation in the other maps by introducing parametric variation (*0* < *x* < *1*) in the phenotypic determinants that were kept fixed during the evolutionary trial. Results reveal that selection on a single map creates significant side-effect phenotypic distributions in the other maps that are not the target of selection. **C** Correlations in the side-effect maps are significant across all parameter values at which the parameter of the evolved map is frozen. *p* = *64* individuals; *n* = *30* replicates, GRN + Multilinear model

To adaptively evolve the “selected map”, an initial heterogeneous population of *p* = *64* individuals is composed by randomly picking each individual (GRN) from the initial random ensemble. According to our previous experiments, each of these individuals exhibits certain “by default” correlation between its maps, yet the average slope of the maps at population level show no particular direction (Fig. 3A). At each evolutionary time step (i.e. within a generation time and for each individual in the population), we introduce variation only in the input associated with the selected map (i.e., genetic, environmental or parental inputs). To make comparisons possible, only one element (e.g. one gene) is varied at a time for each type of input.

In response to the variation on one type of input, each individual develops a set of phenotypes that is compared to an arbitrary (linear) target map to determine the individual’s fitness. In turn, the individual’s fitness determines the likelihood of that individual to contribute to the next generation. Thus, the entire phenotype distribution produced by the selected map is accessible to natural selection (i.e., fine-grained selection). In contrast, the inputs of the non-selected maps were kept fixed (no variation) during simulations, so that these maps remain effectively “invisible” to natural selection (Fig. 3A). Notice that this selection criterion does not select for particular phenotypes, but for particular biases (i.e. trait correlations) in the maps themselves. This procedure allows us to accelerate the weak selection on variation that is found on natural populations, where it is performed indirectly through individual-level selection of phenotypes [9, 10, 33, 34].

In each generation, a number of random point mutations are introduced in the GRN parameters and in the multi-linear coefficients (as in [21], see “Methods”) of the new individuals. Such changes in the developmental architecture may change the way in which organisms respond to the focal inputs, thus creating new selectable variation in the slope of the selected map, and allowing adaptive change in the long-term (< *1000* generations). Once each evolutionary simulation reached a steady state, we assessed if there were any changes in the non-selected maps. We did this by: (1) introducing variation to each of the non-selected maps (one by one), and (2) collapsing the variation for the selected map to a single input value *x* ~ *U(0,1).* In the “collapsed” maps, we deliberately avoid setting *x* = *0* to ensure that our results are due to the lack of variation in the inputs and not to the absence the input itself.

That is, if the selectable phenotypic distribution had been originated exclusively through variation (*0* < *x* < *1*) in the parameters of type “A” (e.g. genetic), and keeping the all parameters of B (e.g. environmental) and C (e.g. parental) types fixed; now all “A”-type parameters are kept fixed and parametric variation is introduced, alternatively, in the “B”-type and “C”-type parameters to quantify the newly arising phenotypic distributions (see “Methods”). This experimental setup guarantees that any observed changes in non-selected maps can be attributed to indirect effects of selection on the selected map.

**Fig. 4:**
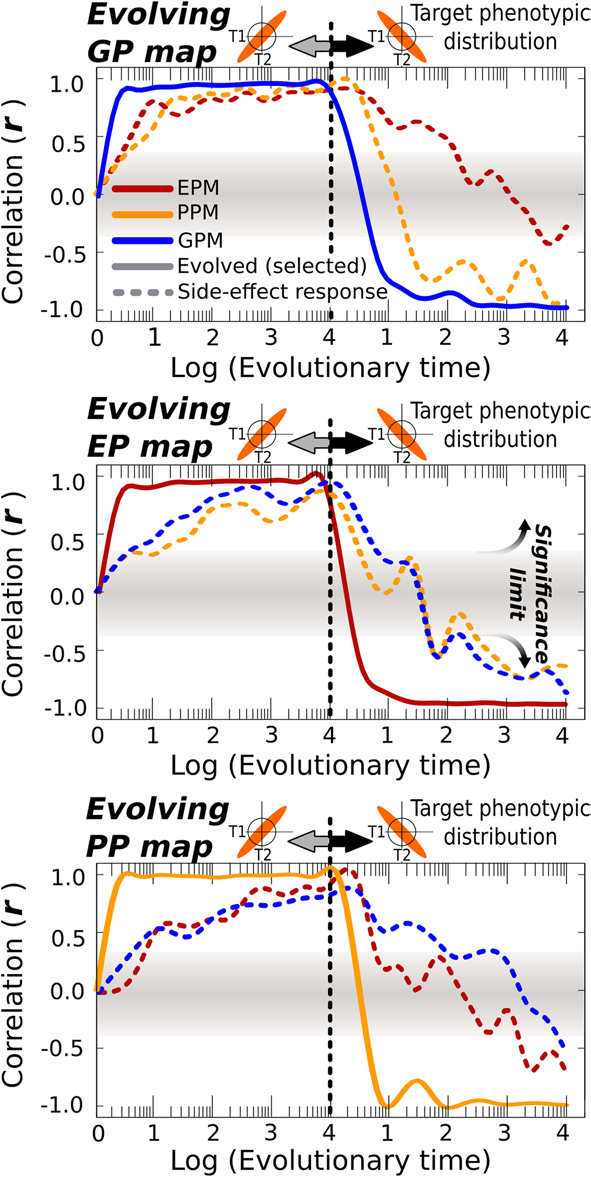
Side-effect phenotypic distributions are able to track shifting targets. These plots show how initially unstructured populations are able to adaptively evolve a specific target distribution (a single map with a defined slope of *S*^*T*^ = *1*, solid lines), which creates as a side-effect correlated phenotype distributions in the other maps (dashed lines). Notice that evolutionary time is plotted here in a Log_(10)_ scale, so that adaptation to the target maps occurs actually very fast (i.e. in a few generations). The middle point corresponds to the steady-state situation shown in Fig. 3. For these plots, the target slope has been shifted to *S*^*T*^ = *−1* at generation *t≈10*^*4*^, showing how the maps that evolve as a side-effect are able to “follow” the one that is being selected. This pattern is similar for every map considered. *p* = *64* individuals; fine-grained selection (identical selective grain for each map); *n* = *30* replicates, GRN + Multilinear model

The results revealed that evolving any one map modifies the other maps as well, introducing in them the same adaptive phenotypic biases as observed in the selected map (Pearson *r* > *0.3*, Fig. 3A). This holds true for every map combination (Fig. 3B) and across the entire range of parameters we tested (Additional file 1: Fig. S2). However, the phenotype biases in the non-selected maps are not as strong (|*r|*< *0.5* in some cases) as in the map under selection (*r* = *0.99*), and exhibit substantial temporal variation, with non-selected maps lagging behind the selected map (Fig. 4). The results in Fig. 3 illustrate the outcome of selecting for a linear map with a slope *S* = *1* in the two-trait morphospace, but simulations with *S* = *−1* or with changing selective pressures yielded similar results (Figs. 4, Additional file 1: Fig. S6).

The correlated evolution of non-selected maps also implies that the ability of a map to adapt may be influenced by past selective events on the other maps. Indeed, the adaptive evolution of any selected map takes longer if (*any* of) the maps had evolved before to match a different target (since evolution has to “undo” the already evolved biases before evolving new ones; Additional file 1: Fig. S7).

### Maps evolve faster under fine-grained selection than under coarse-grained selection

In the previous experiments, each evolving population was allowed to sample a wide range of genetic, parental or environmental inputs in each generation, and selection therefore acted on a wide range of phenotypic outputs. In other words, we assumed a very fine-grained selection. This allowed us to see how adaptation in each individual map would affect the other maps in the hypothetical case where selection is able to effect change in the selected map easily. In natural populations, such fine-grained selection cannot be assumed, so in this section we examine the effects of relaxing this simplifying assumption. To that end, the previous results under idealized, very fine-grained selective scenarios are taken as a “null hypothesis”, and compared against more coarse-grained regimes.

Several studies show that adaptive plasticity readily evolves when selection is fine-grained [35–37], although it is not essential [33]. Whether or not a similar effect occurs for GP and PP mappings is unknown. To address this, we explored the ability of every map to adapt to a target map under different levels of selective grain, ranging from very fine-grained selection (where individuals can experience several inputs within their lifetime) to coarse-grained cases in which there is just one input per generation and this input only shifts every *n* generations.

**Fig. 5:**
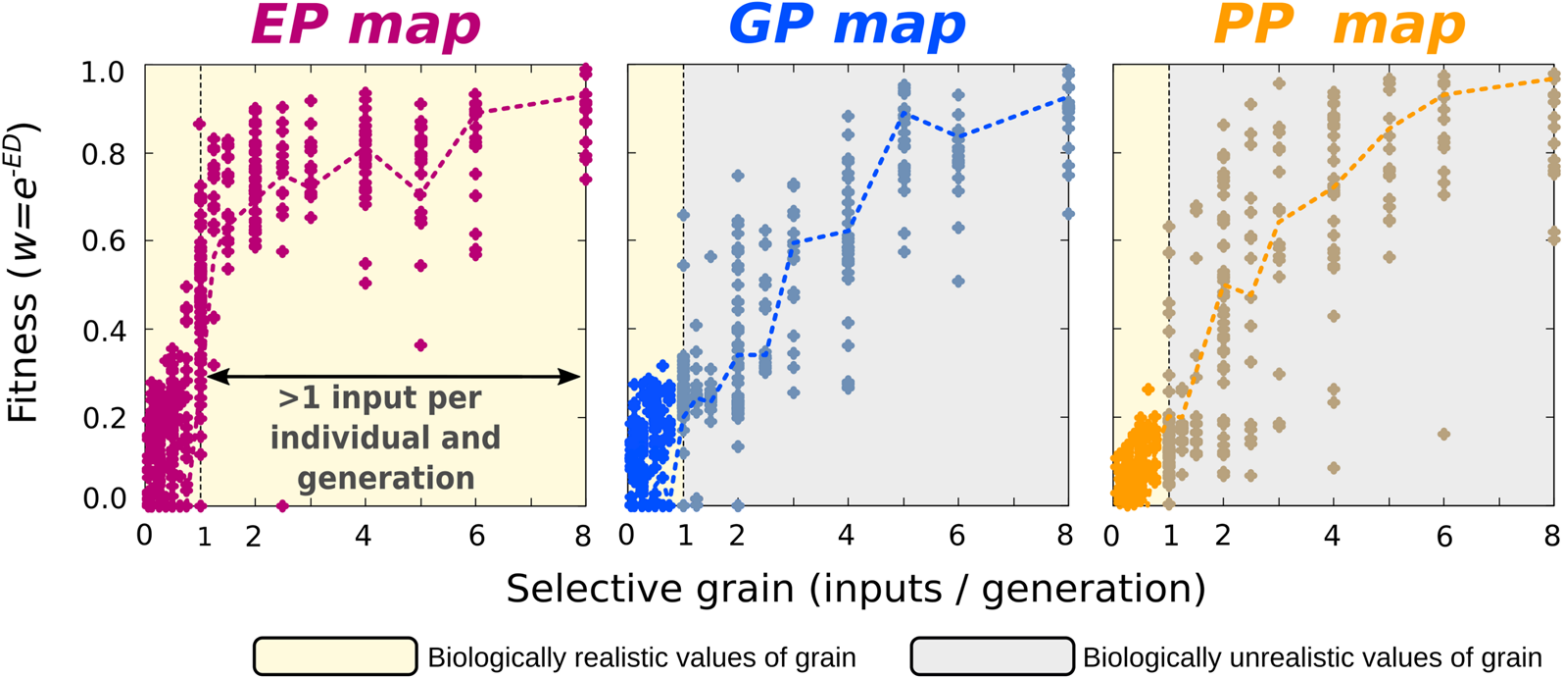
Map evolvability depends on selective grain, and it is maximal for Environment-Phenotype maps. In Figs. 3 and 4, simulations assumed that natural selection could act on the entire map. Since we define selective grain as the average number of parameter-phenotype points that be experienced by a single individual in each generation (and hence “seen” by natural selection, see “Methods”), this corresponds to very fine-grained selection. In this experiment, the assumption about high fine-grainedness has been relaxed. For each level of selective grain, the ability of natural selection to evolve a linear map with an arbitrary slope is recorded as the Euclidean-distance (ED)-based fitness after *t* = *10*^*4*^ generations. Points correspond to individual replicates, and dashed lines to averages over the *n* = *30* replicates. Point colour represents map type. For each replicate, the target map is a linear function of arbitrary non-zero slope. These plots show that the ability to adapt to a target slope increases non-linearly with selective grain, and that maximal efficiency is achieved when selection is fine-grained (> 1), which corresponds to scenarios in which single individuals can experience more than one input per generation. Such high levels of selective grain are typically only attainable for Environment-Phenotype (*EP*) maps (see main text). In the GP and PP-maps, in contrast, such high levels of fine-grainedness (inputs/generation > 1; greyish shadowed areas) represent biologically unrealistic scenarios that can only be revealed by means of in silico experiments. However, understanding the evolutionary dynamics in these “unbiological” regions is fundamental to establish whether the evolutionary dynamics observed in real-world populations arise from differences in the selection grain between maps or from other confounding factor(s) (see main text for a “Discussion”). *p* = *64* individuals, GRN + Multilinear model

As Fig. 5 shows, all maps are in principle equally responsive to strong selection, yet all of them evolve more efficiently under fine-grained selection than under coarse-grained selection. Furthermore, the ability to adapt to the target map escalates sharply around a grain value of 1 (Figs. 5 and Additional file 1: Fig. S9). Under the metrics adopted here (see “Methods”), this is the value where single individuals experience on average more than one input per generation. This implies that it is much easier to evolve a map efficiently, and thus to affect the other maps, if there is within-lifetime variation in the inputs to that map. This disproportionate effect of the most fine-grained screened map on adaptive evolution is observed even when all the three maps are simultaneously selected (Additional file 1: Fig. S8). When maps are not simultaneously selected, but the map under selection is different from the map that *has been* under selection in the recent past (e.g., due to a change in ecological demands), the current evolution of the former will be influenced by the past selective pressures on the later (Additional file 1: Fig. S7). That would make possible, for instance, that past selection for plasticity has an effect on current genetic evolvability.

Besides selective grain, the ability of GRNs to evolve a target map also depends on the complexity of the target map (i.e., how non-linear is the phenotypic response to input variation). Simple (i.e., linear) maps can easily evolve with moderate fine-grained selection (≈2 inputs per lifetime) whilst evolving more complex (i.e., quadratic or cubic) map requires an increasing number of inputs per lifetime (Additional file 1: Fig. S9).

While universal differences in complexity between maps are hard to conceive of (examples of simple and complex responses have been reported for GP, EP and PP maps), there is a clear, widespread difference in the selective grain of the three maps. This arises from the fact that, in most organisms, individuals can experience different environmental inputs during their lifetime but are limited to a single genotype and a single set of parentally inherited initial conditions. As a result, the EP map selection would be most fine-grained, and hence the one most intensely sculpted by natural selection. Because of this asymmetry, the EP map can exercise a stronger influence on the other maps than vice versa (Fig. 5 and Additional file 1: Fig. S8). In other words, while every map can *theoretically* be the leader of adaptive evolution, the logic by which natural selection operates in real-world organisms makes the EP map a prominent driver of evolutionary dynamics. The generality and the evolutionary consequences of this are further discussed in the next section.

## Discussion

Understanding how the processes that generate phenotypic variation interact with natural selection is necessary to explain and predict the course of evolution [3, 5, 7, 38]. While it is easy to understand that any developmental bias aligned with adaptive demands would facilitate adaptation, it is not obvious how these biases originate, nor how they might change or be maintained over evolutionary time. Phenotypic adaptation can precede genetic adaptation, and it has been suggested that plasticity therefore facilitates genetic evolution (reviewed in [23]). However, trying to explain genetic evolvability (i.e., adaptive phenotype biases in response to random mutation) by presupposing the existence of adaptive plasticity overlooks the fact that adaptive plasticity is itself a product of genetic evolution. If explaining adaptive plasticity requires past genetic evolution to have already produced adaptive phenotypic responses to particular environmental cues, this does not help to explain genetic evolvability itself. The idea that plasticity and evolvability are intrinsically linked through development provides a way that selection for plasticity can result in the evolution of genetic evolvability, as studied here. Our aims have been to explore this linkage using mechanistic models of developmental dynamics and thus explore the evolutionary consequences of the relationship between plasticity and evolvability.

Our results show that a concordance between the GP, EP and PP maps is intrinsic to developmental dynamics based on GRNs (with or without selection). Because of this concordance, selection for any map will affect the other maps in an essentially symmetric fashion. However, the efficacy of natural selection in sculpting the different maps is not symmetric because selection for the EP map can be much more fine-grained than selection for the genetic evolvability. Accordingly, selection for plasticity can be much more effective in changing the GRN and genetic evolvability than direct selection for genetic evolvability. Thus, without overlooking the fact that adaptive plasticity is itself a product of genetic evolution, we show how the genotype-phenotype (GP) map can be adaptively shaped by selection for phenotypic plasticity, suggesting that adaptation to environmental variation helps explain the remarkable genetic evolvability of organisms in nature. This finding complements the previous notion that selecting for plasticity may increase the amount of (raw) genetically induced phenotypic variation [21]: we show now that such new induced variation is not only larger but, *in addition*, preferentially aligned with the direction of the adaptive plastic responses.

The results show that the phenotypic effects of genetic and environmental sources of variation are typically similar across a wide range of assumptions. This finding is in agreement with previous research that found similar correlations on other theoretical grounds or for more restricted scenarios, such as selection for developmental robustness to environmental perturbation [16–19, 21, 24]. In a general sense, all these results suggest that organisms exhibiting adaptive plasticity will be expected to have high genetic evolvability, and vice versa. However, the inclusion of the parental (PP) map in our analysis allows us to further generalise our results, suggesting that, by virtue of a shared developmental dynamics, most parametric perturbations in a developmental system will “map” to a similar set of phenotypes. Moreover, since the concordance appears in randomly generated regulation networks, it does not require the concourse of past selection to produce this concordance. Rather, stability analysis revealed that correlations between GP, EP, and PP maps are caused by the structure of the network itself and its associated dynamical properties. Although this result might be expected for statistical models that assume linearity and additivity (e.g., the equality *P* = *GxE* from quantitative genetics, [39]), it is non-trivial for mechanistic models like the ones presented here since these involve non-linear interactions between the inputs. These interactions create a non-uniform space of phenotypic possibilities (a generalised phenotype distribution; GPD) that includes regions of the morphospace that most of the parameter combinations map onto (i.e., phenotypic attractors; [18, 32]), and ‘forbidden’ regions that cannot be attained by any parameter combination. This does not imply that the GPD has an exaggerated robustness preventing the existence of ample phenotypic variation. Rather, these features of the GPD impose strong limitations on the phenotypic variation that is possible, and the shape of the genotype-, environment-, and parental-phenotype maps will be similar since they share the same attractors (i.e. they must be contained within the same variational structure, Additional file 1: Fig. S1). Such complex attractors are an inherent property of many dynamical systems, and can be only revealed by means of mechanistic models or advanced mathematical tools [4, 7, 31, 32].

The reduced degrees of freedom of these shared attractors explain why a population that has evolved a specific (e.g., EP) map will show similar biases in all its maps, even when those have not been selected for. However, this dependence would not make plasticity exercise a disproportionate effect on genetic evolvability unless there was some asymmetry that makes selection for properties of the EP map more efficient than selection for properties of the GP or PP maps. We show that this crucial asymmetry follows from differences in the temporal timescale (‘grain’) of environmental, parental and genetic variation that is input to these maps. Specifically, the variational properties of a map evolve faster when individuals experience multiple inputs, and hence can develop multiple selectable phenotypes, during their lifetime [35–37]. While individuals can experience multiple environments during their lifetime, they do not experience multiple genotypes or initial conditions (e.g., the distribution of phenotypes produced under genetic variation is a property of a family, population or lineage, not an individual). As a result, selection for GP and PP maps should typically be more coarse-grained and less efficient than for EP maps. This general property of natural selection suggests that adaptive EP maps will generally evolve more readily than adaptive GP (and PP) maps, even though they depend on the same developmental dynamics. Notice that the reverse situation (plasticity lagging behind the other maps) might be also possible in some special cases of long-term environmental stasis (e.g., abyssal or deep-soil communities) but, in most evolutionary scenarios, the evolution of phenotypic plasticity would lead the evolution of genetic evolvability much more easily than vice versa.

These results generate predictions that can be tested empirically, for example, by means of experimental evolution. One particularly useful approach would be to select populations in environments of different variability (i.e., selective grain), which should result in populations with different EP maps. The prediction is that the finer the selective grain, the more the structure of the GP map will resemble that of the EP map, which can be tested using mutation accumulation experiments or genetic engineering. Whether or not such changes in the GP map changes the capacity for future adaptation could be tested by exposing populations to new selective regimes that are more or less structurally similar to those that the population was initially adapted to. Other experimental and comparative approaches could also test one or several of the predictions of the relationship between EP, PP, and GP maps (e.g., [12, 19, 40, 41]).

While our results demonstrate that natural selection on phenotypic plasticity would cause the GP map to evolve, they also show that the ability to evolve a certain map is severely limited by the map complexity itself, with complex (e.g., cubic) maps requiring highly fine-grained selection. This would render very complex EP (and GP) maps unreachable by adaptive evolution even in the most fine-grained scenarios [34]. However, complex maps are known to exist, which suggests that other non-selective processes, such as developmental system drift [31, 42], may play an important role in developmental evolution [3, 38]. This possibility is compatible with our results, which simply state that whenever adaptive developmental biases do exist, they will be predominantly the result of past selection for phenotypic plasticity. Furthermore, since each trait needs to maintain its function as other parts of the organism develop and grow, selection for plasticity can be even more fine-grained that expected (our models do not fully capture this developmental dependence because they record the individual fitness after a fixed developmental time).

These results shed light on whether or not plasticity can exercise a predominant role in adaptive evolution, a hypothesis with a long and contentious history in evolutionary biology [13, 21–23, 43–45]. While adaptive modification of environmentally induced phenotypes can make plasticity appear to ‘take the lead’ in evolution without any link between plasticity and genetic evolvability [13, 41], the evolutionary change in the GP map caused by adaptive plasticity suggests that evolution is particularly likely to proceed where plasticity leads. Over longer timescales, this process provides a biologically plausible mechanism for the internalisation of environmental information, resulting in developmental biases whose structure ‘mirrors’ the structure of the selective environment [46], and thereby facilitating further adaptations through genetic modification of environmentally induced phenotypes [8, 13, 21, 33]*.* Without denying the importance of other (adaptive and non-adaptive) processes (e.g. [42]), this constitutes a strong argument for a role of plasticity in shaping the path of evolution.

Note that our results do not imply that phenotypic plasticity is a leader in the sense that it arises without genetic evolution. Rather, we suggest that the *genetic evolution of* plasticity comes first, and this causes (by common cause of developmental mechanics) the evolution of genetic evolvability along the phenotypic directions that were initially plastic. This account is fully compatible with natural selection on genetic variation as a prime-mover in evolution and yet suggests a meaningful sense in which phenotypic plasticity comes before genetic evolvability.

## Conclusions

In summary, our work reports a previously ignored process that explains how development can turn random genetic variation into adaptive phenotypic variation. Such a process, which entails an enhanced scope for adaptive evolution, emerges naturally from a combination of two different and well established phenomena. The first is that development makes the phenotypic consequences of genetic and environmental changes generally similar. Therefore, selecting for particular responses to environmental variation (i.e., phenotypic plasticity) can affect the way organisms respond to genetic mutations (i.e., their genetic evolvability) and vice versa. The second is that natural selection is more efficient in evolving phenotypic plasticity than genetic evolvability (making plasticity commonly adaptive). The developmental linkage allows these adaptive responses to environmental variation to be transferred to the genotype–phenotype map, thereby facilitating the generation of adaptive phenotypic variation through genetic change. These findings suggest that organisms can evolve in the long term by exploiting changes to the GP map that were originally evolved for their capacity to adaptively respond to environmental variation in the short term.

## Methods

### Correlations between maps

A large ensemble (*n* = *10*^6^) of random GRNs was created by setting the probability of non-null genetic interactions to *p(B*_*ij*_* ≠ 0)* ~ *U(0,1).* In addition, we uniformly sampled the GRN space of networks between 3 and 24 genes, (*N*_*g*_ ~ *U(3,24)*) so that a variation in GRN size and connectivity were represented (by virtue of the central limit theorem, average GRN size and connectivity are, respectively, *N*_*g*_* ≈ 14.5* and *p(B*_*ij*_* ≠ 0)≈0.5*). Environmental inputs were also assigned randomly, being each element of the *G* vector drawn from an exponential distribution *E*_*i*_ ~ *Exp(λ* = *1).* This ensures that just some environmental inputs are able to interfere effectively with developmental dynamics (otherwise the effect of environment on GRN dynamics would be too large). The same ensemble was used for the three models. Model-specific elements were randomly drawn: *Z*_*ij*_ ~ *U(−1,1)* for the multilinear coefficients of the mixed model, and *D*_*i*_ ~ *U(0,1)* for the diffusion rates of the lattice model. For each random GRN, parameter-to-phenotype maps were generated through systematic (parametric) perturbations in each of the GRN elements. The element perturbed was randomly chosen for each replicate (*n* = *30*) and given values from 0 to 1 at 0.1 intervals (in this work, the input values of the different maps are of similar magnitude, an idealisation that allows us to compare the evolutionary properties of the different maps). During these perturbations, GRN topology was always held fixed. Perturbations in *B*_*ij*_ were conceptualised as genetic changes; in *G*_*0*_ as changes in the initial conditions (i.e., parental effects); and in *E*_*j*_ or *D*_*i*_ as environmental changes (Fig. 1). That way, the systematic perturbation of each element generated 10 different phenotypes that were recorded in a two trait morphospace, constituting a map (GP map, PP map or EP map, respectively). Note that our “maps” are not maps in a formal mathematical sense because they do not retain the univocal relationship between the inputs and the outputs. However, they allow us to compare different phenotypic distributions whose inputs have different units and magnitudes.

We focus on two-trait phenotypes because they embody the minimal multivariate system where associations between traits can be found (see below for simulations involving more than 2 traits). In the basic, 2-trait system, maps were compared, two by two, using two measures of map-to-map similarity. The first is a coarse-grained measure: Pearson’s *r* correlation between the two linear slopes in the phenotypic morphospace (Fig. 2). In order to take into account negative and close-to-zero slopes, the original slope values were transformed to *S*_*a*_ = *sgn(S)·Log(1* + *S)*, so that negative values correspond to negative slopes, and not to *0* < *S* < *1* (therefore, the radially symmetric distribution of points around the origin (0,0) observed in in Fig. 2A suggests that individual trait-trait correlations across maps have similar likelihood of being positive or negative). Two maps *a* and *b* were said to be correlated or uncorrelated depending on their sectorial position in this correlational (*Sa,Sb*) space: *corr(a,b) ↔|tan-1(Sa/Sb)-π/4|*≤ *π/12, anticorr(a,b) ↔|tan-1(Sa/Sb)* + *π/4|*≤ *π/12,* and not correlated otherwise (Fig. 2B). The second, fine-grained measure is the Euclidean distance (*ED*_*a,b*_) between maps *a* and *b* (Additional file 1: Fig. S3):

5 $$ED_{a,b} = \sqrt {\sum\nolimits_{j = 1}^{10} {} \left( {T_{aj1} - T_{bj1} } \right)^{2} + \left( {T_{aj2} - T_{bj2} } \right)^{2} }$$

where *T*_*ijk*_ is the value of trait *k* in the *jth* phenotype of map i. As Additional file 1: Fig. S3 shows (panel A), *ED*_*a,b*_* α|S*_*a*_*/S*_*b*_*|*. As a proxy for map complexity (*C*_*a*_) we use the sum of the squared residuals of each map with respect to its linear regression, which simply measures the quality of the fit of the map to the linear function *y* = *S*_*a*_*·x;* where *S*_*a*_ is the slope of the map itself. While more mathematically precise measures of map complexity can be envisioned (e.g. Bayesian Information Criteria, Fourier analysis), our measure intuitively captures the notion that the more a map departs from a perfect line the more complex it is:

6 $$C_{a} = \sum\nolimits_{i = 1}^{10} {\left( {T_{i2} - T_{i1} S_{a} } \right)^{2} }$$

where *T*_*ij*_ is the value of the trait *j* of the *i*th point (phenotype) of the map considered. For this analysis maps were re-scaled to (*0* < *T*_*ij*_ < *1*) values in order to avoid size-effects on the map complexity (otherwise the squared residuals of maps with large phenotypic values would result in artefactually higher complexity) (Additional file 1: Fig. S3B). We assess the effect on map-map similarity (slopes and map complexities) of GRN size (*N*_*g*_) and connectivity *p(B*_*ij*_* ≠ 0)* (Additional file 1: Fig. S4), but not of GRN topology itself as this is beyond the scope of this work (for a discussion on this see [30, 31]).

Finally, we assessed the possible effect of the number of traits considered (*Nt* > *2*) on the map-to-map comparisons, using three independent methods. In Method-1 (Composited traits), slopes and (Pearson-r) correlations are calculated as in the two-trait (T1–T2) basic model but assuming, instead, that the first trait (T1) is a composite trait containing (sub)traits 1 to *Nt/2,* and that the second trait (T2) is a composite trait containing (sub)traits *Nt/2* + *1* to *Nt*. In Method-2 (Averaged slopes), correlations between maps are calculated using the average (linear) slope (Ŝ) of each map. Each average slope Ŝ is in turn calculated using the slopes of all possible two-trait combinations from the set of *Nt* traits:

7 $$\hat{S} = \left( {\begin{array}{*{20}c} {N_{t} } \\ 2 \\ \end{array} } \right)^{ - 1} \sum\nolimits_{i = 1}^{{N_{t} }} {\sum\nolimits_{j = 1}^{{N_{t} }} {S\left( {T_{i} ,T_{j} } \right)} }$$

where the left parenthesis indicates the number of trait combinations satisfying T1 < T2. Method-3 (Euclidean-based comparison) follows Eq. 5, but generalize the measure to a *Nt*-dimensional space:

8 $$ED_{a,b} = \sqrt {\sum\nolimits_{j = 1}^{10} {\sum\nolimits_{k = 1}^{{N_{t} }} {N_{t}^{ - 1} \left( {T_{ajk} - T_{bjk} } \right)^{2} } } }$$

Independently of the method used, all simulations of this experiment satisfy *N*_*g*_ > *Nt* (otherwise high artefactual correlations would arise from the same gene controlling several traits). The results (summarized in Additional file 1: Fig. S10) suggest that increasing the number of traits does not compromise the map-to-map correlations described throughout the paper (although specific trends may depend on the method used for multi-trait comparison).

### Numerical integration

Following [30, 31, 38], all the differential Eqs. (1 and 4) were numerically integrated using the Euler method (*δ*_*t*_ = *10*^*–3*^).

### Control experiments

Two control experiments were set up to better understand the causes of the observed correlations between slopes *S* and map complexities *C*. In the first, with a probability *p* = *{0.1,0.2,…1},* GRN topology was changed as *M*_*ij*_* →|M*_*ij*_*−1|* and the GRN input values as *x → x* ~ *U(0,1)*. Then, correlations were recorded between the maps arising from the unperturbed GRN (*S* and *C*) and the randomized ones (S* and C*, Additional file 1: Fig. S4). In the second control experiment, we used a set of randomly generated mathematical functions (polynomials *f(x)* with known degree *deg(f)* ≤ *4*) as a null, non-generative space in which we could test whether the observed correlations between map complexities are a general property of any mathematical function rather than a biologically relevant phenomenon (argument and value of *f(x)* are considered to correspond to the traits *T*_*1*_ and *T*_*2*_). A discrete “mapping” was created by assigning ten values to *x* = *{0.1,0.2,…1}*, and then calculating the corresponding *y*-values (Additional file 1: Fig. S5):

9 $$y\left( { \approx T_{2} } \right) = \sum\nolimits_{i = 0}^{4} {R_{\left( i \right)} x\left( { \approx T_{1} } \right)^{i} e^{ - i} }$$

where *R* is a vector of random numbers *R(i)* ~ *U(0,1)* and *e*^*−i*^ a corrective token that devalues the high-degree terms of the function, ensuring that polynomials of different degrees are equally represented. If necessary, *y-*values were rescaled to (*0* < *y* < *1*, as in Additional file 1: Fig. S3), so that the map complexity of the the function was measured under the same conditions as for GRNs.

### Evolving maps

Several of our experiments involve the adaptive evolution of a map: a population of *p(*= *64)* non-recombinating (haploid) individuals picked from the random ensemble evolves in a mutation-selection-drift scenario until a map with a target slope *S*^*T*^ is encountered, or until a maximum number of *t*_*max*_ = *10*^*5*^ generations is reached (non-overlapping discrete generations are used in all simulations). Arbitrarily, *S*^*T*^ is set to *S*^*T*^ = *1* (other choices do not alter the results, see Additional file 1: Fig. S6). With a rate of 0.04 (*≈1/N*_*gmax*_) per element and generation, point mutations are introduced in the matrices encoding the topology and interaction strengths of the GRN: *B*_*ij*_* → B*_*ij*_ + *ξ (ξ* ~ *N(μ,σ); μ* = *0, σ* = *0.01)* and *M*_*ij*_* →|M*_*ij*_*−1|*. The same rate of change is applied to the coefficients of the multi-linear model used in the evolutionary simulations, which mutate as *Z*_*ij*_* → Z*_*ij*_ + *ξ (ξ* ~ *N(μ,σ); μ* = *0, σ* = *0.01)*. Such a mutation process is applied to every individual within the population at every generation. The fitness of each individual *W*_*i*_ is calculated on the basis of its ability to create a map similar to the target one (not on the basis of a single phenotype). Thus, each individual in each generation is exposed to 10 different inputs in one of its GRN elements (the element depends on the map being evolved), and its slope *S*_*i*_ in a *T*_*1*_*–T*_*2*_ morphospace recorded and compared to the target slope *S*^*T*^. This algorithm is formally equivalent to an inter-generational variation in the inputs [33–35]. The similarity with the target slope determines the individual’s fitness and, in turn, the probability of each individual to contribute to the next generation:

10 $$W_{i} = e^{{ - \left| {S_{i} - S^{T} } \right|}}$$

Some of our experiments involve different levels of selective grain on the maps, which has two different components: intra-generational (i.e., how many different inputs (or points of the whole map) can the population experience in a single generation) and inter-generational (i.e., how often these inputs change, which can be conveniently expressed as the number of generations between changes in the input values). For the sake of simplicity we collapse these two components in a single composite measure of fine-grainedness as inputs/generation (Figs. 5, Additional file 1: Figs. S8 and S9). Since slopes alone cannot account for the number of points in a map, the fitness is now calculated as:

11 $$W_{i} = e^{{ - ED_{{mapi,map^{T} }} }}$$

where *ED*_*mapi,map*_*T* is the Euclidean distance, point by point, between the individual’s map (*mapi*) and the target map (*map*^*T*^), as described in Eq. (5).

## Supplementary Information

**Additional file 1.** Supplementary figures.

## Data Availability

A basic version of the code used to perform the virtual experiments (written in Fortan95 language), along with some instructions and pertinent datasets have been deposited in the open dissemination research data repository Zenodo, and can be found at https://doi.org/10.5281/zenodo.5607704. All the data supporting the findings of this study can be generated/reproduced using this code and the information contained within the article and its additional information file.

## Abbreviations

GRN: Gene regulatory network
GP map: Genotype–phenotype map. It represents the relationship between phenotypic and genetic variation
EP map: Environment-phenotype map. It represents the relationship between phenotypic and environmental variation
PP map: Parental-to-phenotype map. It represents the relationship between phenotypic variation and variation in the parentally inherited (epigenetic) factors
*N*_*g*_: Number of transcription factors in the core GRNs
*N*_*e*_: Number of environmental factors
*T*_*i*_: Phenotypic trait *i* (the nature of the traits varies with the model used)
*G*: vector (of size *N*_*g*_): Its elements *g*_1_*,…,g*_*Ng*_ represent the concentration of each transcription factor in the core GRN
*G*_*0*_: vector: It accounts for the initial state of* G* at the beginning of development
*G**: vector: It is the normalized (0–1) expression of *G*
*E* vector (of size *Ne* (= *N*_*g*_)): Its elements *E*_*1*_*,…*,*E*_*j*_ represent the effect of the environmental factor *j* on the gene *j*
*B* matrix (of size * N*_*g*_* x N*_*g*_): Its elements *B*_*ik*_ represent the effect of gene *k* on the transcription of gene *i*
*M * matrix (of size * N*_*g*_* x N*_*g*_): Encodes the GRN topology, so that the interaction *B*_*ik*_ is only active if *M*_*ik*_ = *1*
*t*_*dev*_: Number of developmental iterations
*K*_*M*_: Michaelis–Menten coefficient
R: Ramp Function (*R(x)* = *x, ∀ x* ≥ *0* and 0 otherwise) used to calculate GRN dynamics
*μ*_*i *_ decay term: Specific degradation rate of the transcription factor *i*
*ξ* term: Gaussian noise
*Z* matrix (of size *N*_*g*_ × *2*): Its elements *Z*_*ij*_ represent the linear contribution of the gene *j* gene to the trait *i* (Mixed model)
*N*_*c*_: Number of (non-motile) cells in the lattice model
*D*_*i*_: Specific diffusion rate of the transcription factor (morphogen) *i* (used only in the lattice model)
*S*_*a*_: Linear slope of the map *a*
*S*^*T*^: Target slope (only used in evolutionary experiments)
*Ŝ*: Average slope between maps
*ED*_*a,b*_: Euclidean distance between the maps *a* and *b*
*C*_*a*_: Complexity (i.e. non-linearity) of the map *a*
*p*: Population size (only used in evolutionary experiments)
*W*_*i*_: Fitness of the individual *i* (only used in evolutionary experiments)
