# Supplementary material for: Development and selective grain make plasticity 'take the lead' in adaptive evolution
Source: BMC Ecol Evol. 2021 Nov 20;21:205. doi: 10.1186/s12862-021-01936-0 (PMC8605539; doi:10.1186/s12862-021-01936-0)
Supplement: Supplementary file 1 — Additional file 1. Supplementary figures. [file 12862_2021_1936_MOESM1_ESM.pdf]

Additional file 1.

Accompanying the article:
“Development and selective grain make plasticity ‘take the lead’ in adaptive evolution”
By: Miguel Brun-Usan*, Alfredo Rago, Christoph Thies, Tobias Uller, Richard A. Watson.

Additional Figures S1-S8

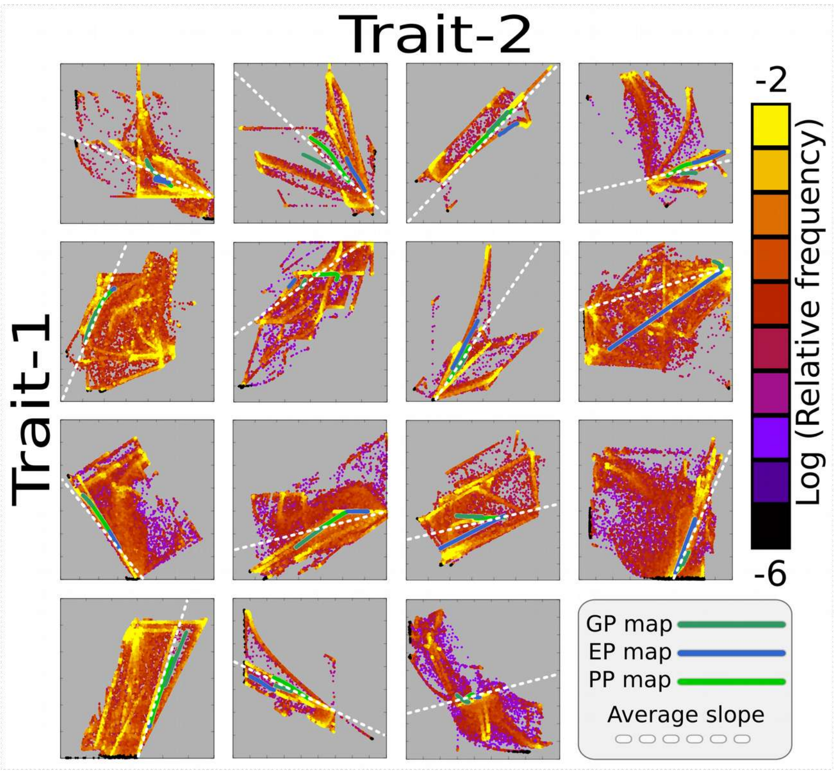

Fig. S1. General Phenotypic Distributions (GPDs). A general phenotypic distribution (GPD) is the total amount of phenotypic variation that a given developmental mechanism (with a fixed GRN topology) can produce under systematic variations of all their phenotypic determinants (i.e. genetic, parental and environmental perturbations). The concept of GPD explicitly expands the previously proposed concept of “variational properties” (See Ref. [13]) in order to incorporate parental variation. This figure shows 15 different GPDs, each one corresponding to a different but representative GRN of our random ensemble. For each one, instead of varying one single GRN element at a time using ten pre-established values (0 to 1 at 0.1 intervals), we vary all of them simultaneously and in a more continuous manner (100 random values $0<x<1$). That is, instead of $n=30$ phenotypes (10 genetic+10 parental+10 environmental perturbations), we get $n=10^6$ (100x100x100) potential perturbations (and phenotypes) per GRN. Each point in these panels corresponds to one of these 10^6 potential phenotypes in a T_1-T_2 morphospace (scaled to fit the minimum and maximum T_1-T_2 values of the GPD). The colour represents the density of points: regions with high density (calculated on the basis of a 100x100 grid) are coloured in yellow, and regions with rare phenotypes are coloured in purple. Notice that the GPD has two levels of structure: One is the set of developmentally possible phenotypes (the occupied area of the morphospace). As the panels show, this is often discontinuous and non-isotropic. The other is the different likelihood of the different phenotypes within this region. More probable phenotypes show up as yellow regions and ridges. This two-level structure of the GPD suggests that development creates correlations between the maps (dark green, light green and blue lines) because the maps most likely contain phenotypes belonging to the ridges of maximal phenotypic probability (which are often parallel or sub-parallel). Dashed white lines are the average slope of the GP, EP and PP maps (dark green, blue, and light green lines, respectively). All are from the GRN + Multilinear model.

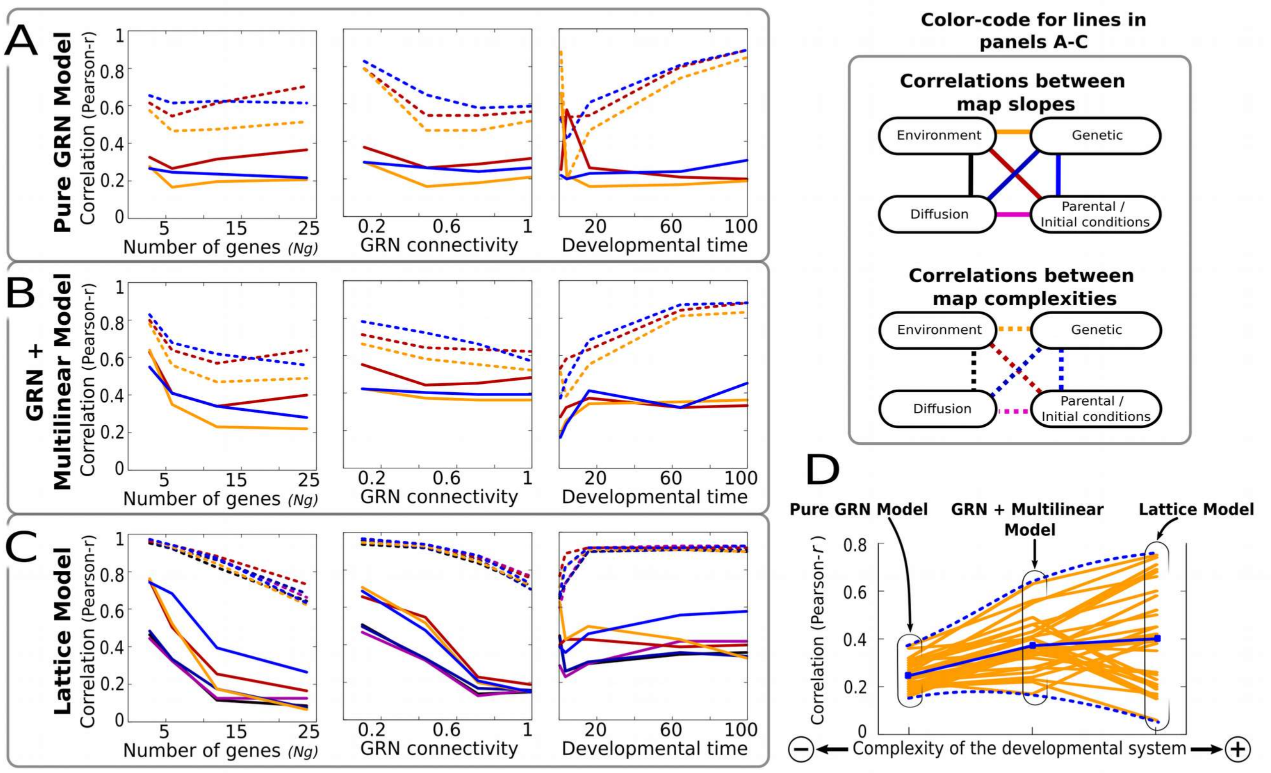

Fig. S2. Map-to-map correlation are robust across the different models considered. A large ensemble ($n=10^6$) of random developmental mechanisms was generated by creating a random GRN (as in Fig. 2, see also Methods) and by assigning random values ($U\sim(0,1)$) to the remaining model-specific parameters (see Methods). For each of these mechanisms, systematic parametric variations were introduced to each of the GRN elements (genetic, initial conditions, environmental inputs). In the case of the lattice model, variation was also introduced in the diffusion rates D_i. Each perturbation on an element generates a collection of phenotypes (a map) in a two-trait morphospace, characterised by a linear slope S and a map complexity C. The panels show the correlations (Pearson's r) between pairs of slopes (solid lines) and pairs of map complexities (dashed lines) for the three models considered: the pure GRN Model (A), the GRN + Multilinear model (B) and the lattice model (C). The schemas on the top right corner assign a specific colour to each map-map pair (notice that the three models are readily comparable because they map onto the same two-trait morphospace and have similar ranges in the parametric variation). For each model, the randomly generated mechanisms have been sorted according to their number of genes (N_g) and GRN connectivity (proportion of non-zero elements in the matrix B), to see how these topological features affect the map-to-map correlations (left and central panels). In addition, for each mechanism the correlations have been recorded over a different number of developmental iterations (right panels). Overall, this figure shows that Pearson correlations are generally significant ($r>0.3$) across the different models considered and under different topological and developmental constraints, although they tend to be weaker as GRN size or connectivity increase. (D) Since the different mechanisms were essayed using the same set of ($n=10^6$) GRNs, we assess the effect of an increased model complexity in the correlations. Yellow lines represent the different slope-slope correlations resulting from each model under the different GRN features considered (blue line is the average). For instance, one yellow line may represent correlations (under the three models) for $Ng=24$ and a GRN connectivity of 0.5; other may represent the same but for a $Ng=12$ and a GRN connectivity 0.8, and so on. This plot shows how adding extra layers of complexity (e.g., a multi-linear layer or multi-cellular reaction-diffusion processes) to a basic GRN affects the map-to-map correlations.

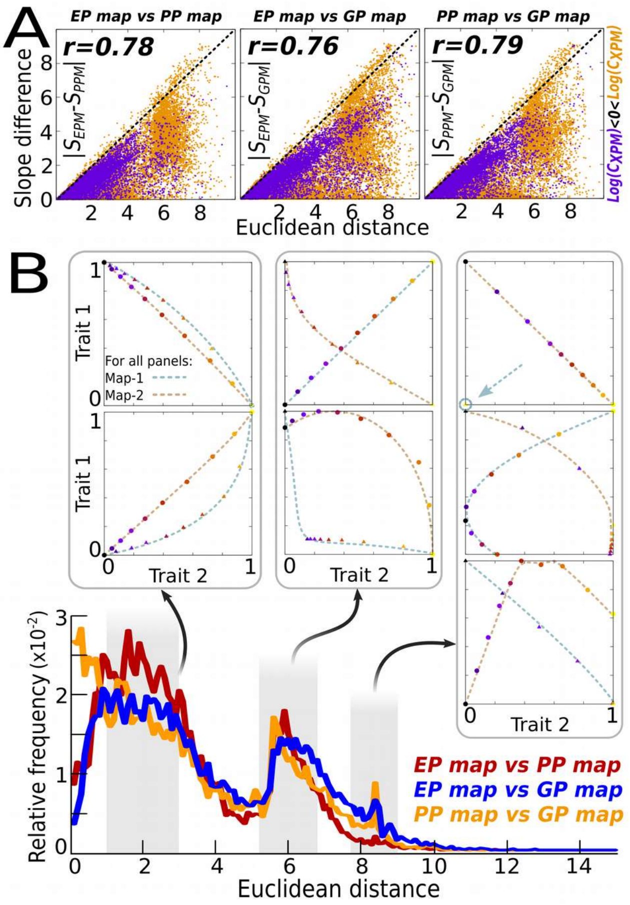

Fig. S3. Correlation between maps is robust when using fine-grained measures of phenotypic distances. In addition to slope correlation (a coarse-grained measure of phenotypic distances) between maps, we have also measured map-to-map similarity using the Euclidean distances (*ED*) between them (a fine-grained measure of phenotypic distances that takes into account distances between pairs of individual phenotypes). (A) *ED* between paired maps for each GRN in our random ensemble are compared with the slope similarity (absolute value of slope differences) between maps. We proceed that way because there is no *a priori* null expectation about which would be the minimum *ED* value above which the similarity would be significant. The three panels show that slope similarity strongly correlates (Pearson's $r>>0.3$) with the Euclidean distance between any pair of maps. This implies that the former coarse-grained measure can be confidently used as a proxy of map similarity, as it has been used in most plots. In addition, we have detected that complex parameter-to-phenotype maps ($Log(C)>0$), yellowish dots) have a much noisier relationship between slope differences and Euclidean phenotypic distances (*EDs*) than simpler maps ($Log(C)<0$), purple dots). This is probably due to the fact that, although complex maps tend to have larger *EDs* between them, their linear slopes tend to be small, which explains the great scattering of yellow dots below the diagonal. (B) The whole random ensemble is analysed here in terms of *EDs* alone. The frequency plot shows a skewed multimodal distribution where the majority of maps have moderate $EDs{\approx}2$ between them. This correspond to cases where the maps being compared show relatively simple, monotonic and smooth trait covariation (see example maps above in a normalized T_1-T_2 space). A second peak in frequency happens around $EDs{\approx}6$ account for the maps that, although still showing monotonic trait covariation (see above), exhibit flipped slopes (a situation that is statistically more common than no-correlated maps, see Fig. 2B). This third peak ($EDs{\approx}8.5$) contains either highly non-linear maps or pairs of maps where one them shows no parametric sensitivity (a point in the T_1-T_2 space, see top right panel). This third peak roughly corresponds to the bulk of yellow off-diagonal points shown in (A). $n=10^6$. GRN + Multilinear model.

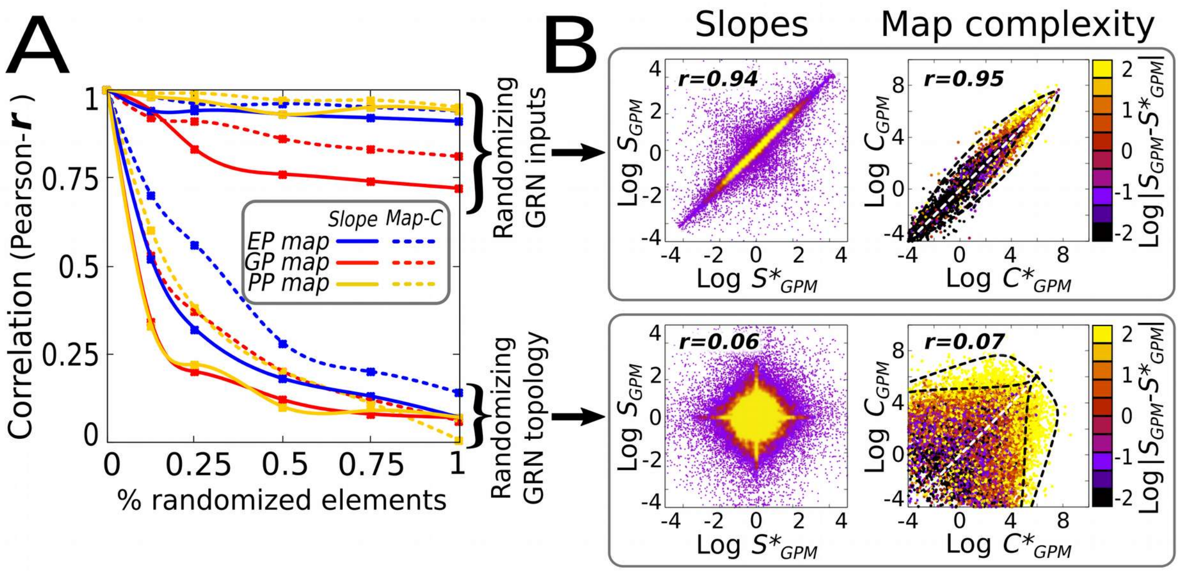

Fig. S4. GRN topology as the main contributor to phenotypic correlations. The figure summarizes a stability test aimed to better understand the causal origins of the map-to-map correlations observed in Fig. 2 (specifically, if correlations arose from the developmental mechanism itself or from similarities on the input parameter values). In order to discriminate between these two causal factors, we gradually randomize both (with a probability $p=\{0.1,0.2,...1\}$; the GRN topology was changed as $M_{ij} \rightarrow |M_{ij}-1|$ and the GRN input parameters as $x \rightarrow x \sim U(0,1)$) while recording if the correlations between maps were still retained or not. Notice that here correlations are established between maps arising from the unperturbed (elemental) GRN (slopes S and map complexities C) and the perturbed, randomized ones (S^* and C^*). (A) For every GRN in our random ensemble, correlations are robust against input randomization but fall dramatically to non-significant values ($r<0.3$) when the GRN topology is allowed to change parametrically. The observed pattern is very similar for both slopes (solid lines) and map complexities (dashed lines). This insensitivity to the randomization in the input values (see Methods) suggests that they are not causally involved in the correlations observed in Fig. 2A. On the contrary, correlations rather show a strong dependence with the GRN topology, which embodies the dynamical developmental system that creates the phenotypes. (B) Each point plotted in (A) represents a correlation measured in a two-dimensional space for slopes S or map complexities C (As in Fig. 2A). We provide, as an illustrative example, two of these spaces corresponding to maps which arise from genetic perturbations (spaces for other types of perturbations are very similar, Fig. 2A). In the upper row, all the maps emerging from a set of genetic inputs $x=\{0,0.1,0.2,\ldots,1.0\}$ are compared with those emerging from maximally randomized genetic inputs $x \sim U(0,2)$. The limited scattering suggests that a given GRN produces almost identical GP maps irrespective of the inputs used. Below, using the same set of genetic inputs $x=\{0,0.1,0.2,\ldots,1.0\}$ and the same GRN ensemble, maps emerging from elemental GRNs and from perturbed GRNs whose topologies have been fully randomized ($M_{ij} \rightarrow |M_{ij}-1| \forall\ M_{ij}$) are compared, showing that, despite having identical inputs, they produce totally unrelated phenotypes. r stands for Pearson's correlation. Yellow areas in S_{GPM} *vs* S^*_{GPM} spaces (left plots in B) contain 90% of GRNs. $n=10^6$. GRN + Multilinear model.

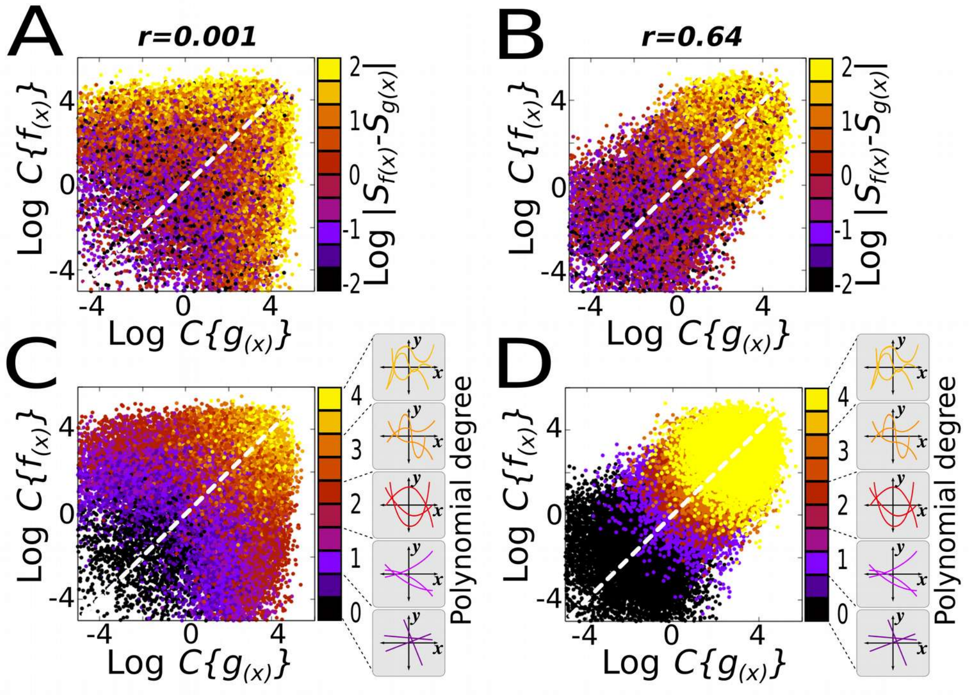

Fig. S5. Geometry alone does not explain the observed positive correlation between map complexities. The aim of this experiment is to assess whether the observed correlation between map complexities (Fig. 2C) is a general feature mathematical functions or not. In order to do this, we analysed the relationship between function complexities (measured the same way as map complexity *C*, see Methods) in a large ensemble of random polynomial equations of known degree (*deg(f)≤4*), where the *x,y* variables of *f(x)* are considered to correspond to the T_1 and T_2 traits (see Methods). (A) When two functions *f(x)* and *g(x)* are randomly picked from the ensemble, no correlation between their map-complexities is observed. Colours express absolute differences between slopes (As in Fig. 2C). (B) A positive correlation between map complexities only appears when the functions to be compared are first sorted according to their complexity class, that is when *deg(g(x)):=deg(f(x))*. (C) The same as A, but colours now represent differences on the degree of the functions (|*deg(f(x))-deg(g(x))*|) rather than differences between slopes. (D) The same as A, but colours now denote the degree of the functions *deg(f(x))* (since functions have been previously sorted, it is the same as *deg(g(x)*). Notice than in B and D, the correlation only appears as an inter-class effect simply because the function complexity correlates with the degree of that function (*deg(f(x)αC{f(x)}*), but it is absent within the same class functions. This suggests that development creates correlations between the map complexities by restricting the number of developmental outcomes to well-defined and mechanism-specific possible general phenotypic distributions (GPDs, Fig. S1). $n=10^6$.

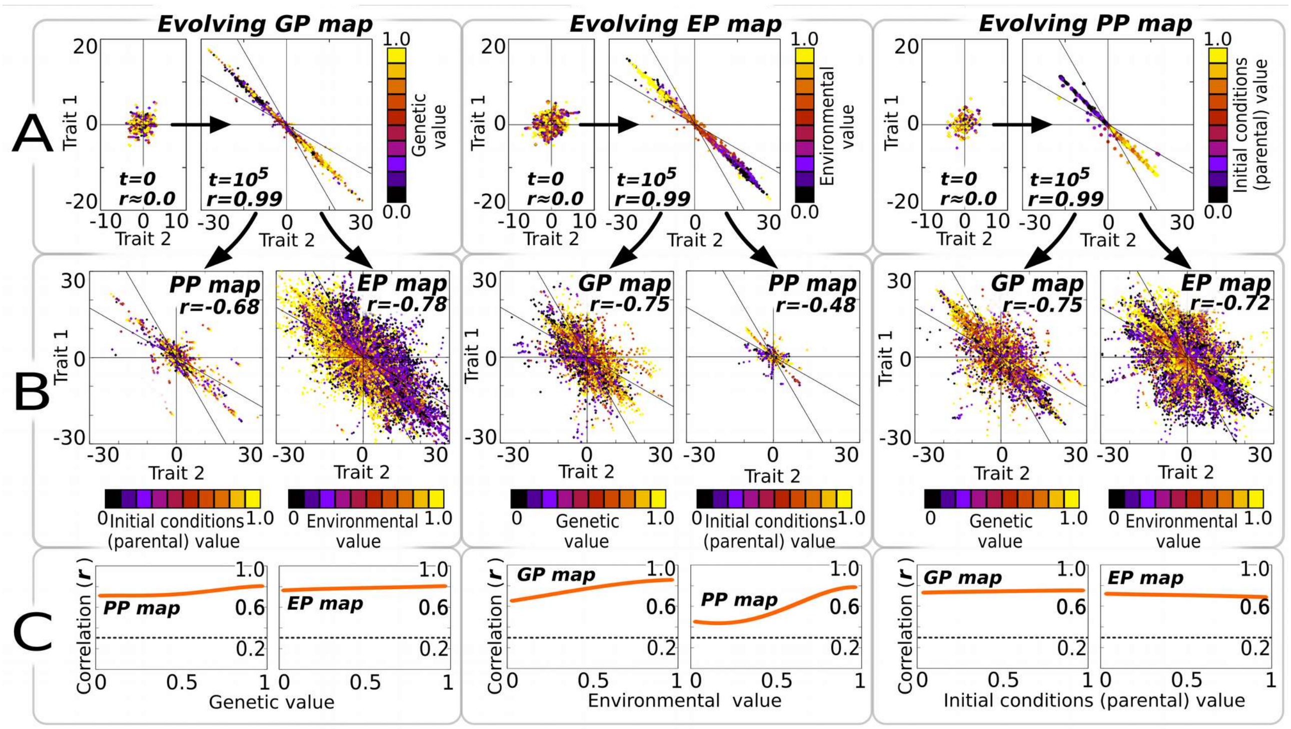

Fig. S6. Using negative slopes (anti-correlated traits) as a target does not change the general results of map evolution. This figure reproduces the same results of Fig. 3, but in this case a target map with a slope *Sx=-1* (instead of *Sx=1*) has been used in the simulations. A comparison between both figures reveals that results of the experiments involving adaptive evolution of maps are very similar when using different (linear) targets. As in Fig. 3, a population whose individuals initially exhibit no particular phenotypic distribution in *t=0* (A, small panels) is evolved to fit a target phenotypic distribution using as an input just one kind of phenotypic determinant. In each generation, one individual is exposed to 10 different values ($0<x<1$) of this phenotypic determinant, thus producing a set of ten potential phenotypes whose slope in a T_1-T_2 space is compared with the target to evaluate the individual's fitness W_i, see Methods). After 10^5 generations in a mutation-selection-drift scenario (where other sources of phenotypic variation are frozen), population has a narrow phenotypic distribution in the evolved map (A, big panels). Then, we uncover the hidden variation in the other maps by i) freezing the variation in the parameter that generated the evolved map to an intermediate value and ii) introducing parametric variation ($0<x<1$) in the other phenotypic determinants (those that were kept fixed during the evolutionary trial). (B) Results reveal that evolving a single map adaptively creates significant side-effect phenotypic distributions in the other maps that are not the target of natural selection. (C) Correlations in the side-effect maps are significant irrespective of the value at which the parameter of the evolved map is frozen. *p=64* individuals; *n=30* replicates. GRN + multilinear model.

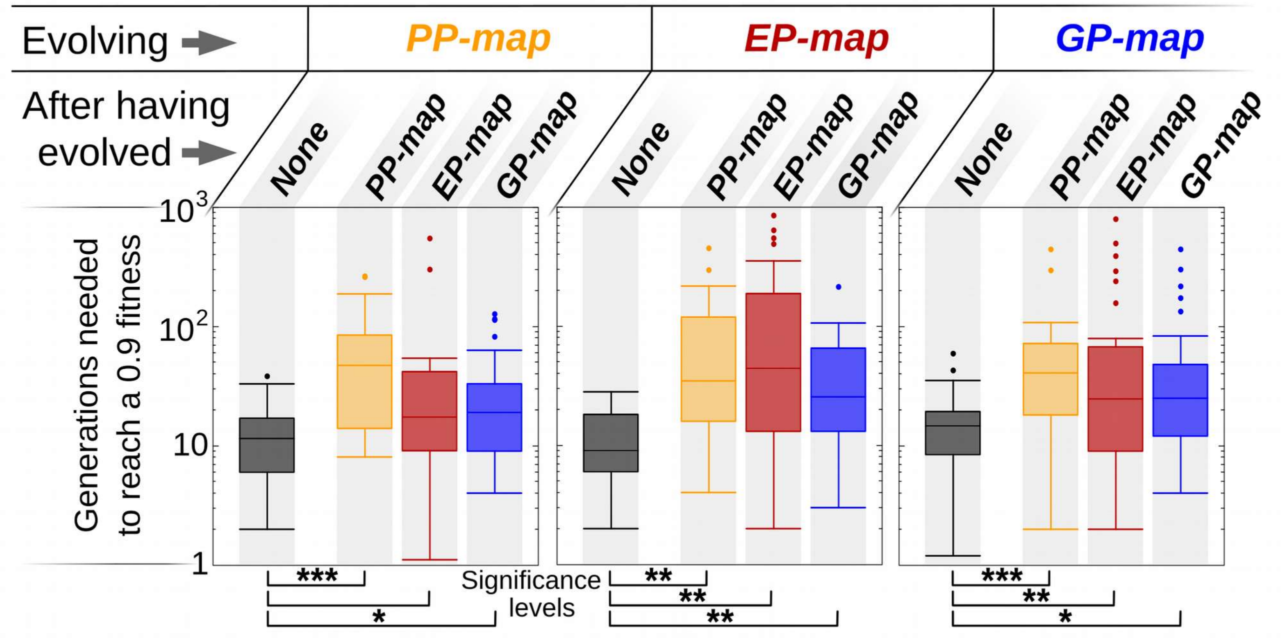

Fig. S7. The response of a map to natural selection is influenced by past selective events on each maps. A population (*p=64*) whose individuals initially exhibit no particular phenotypic distribution in *t=0* is evolved using a *S=+1* slope as a target until an average population fitness of $\dot{W}=0.9$ is reached. For each map, the (Log) number of generations required to reach such a $\dot{W}=0.9$ is recorded (dark grey boxplots). This represents an (Null-model) evolutionary scenario with no previous selective history on the maps (the individuals were randomly chosen from the random ensemble), and consequently the populations evolve the target maps rapidly (in a few dozens of generations). Then, those populations are adaptively evolved using a different (*S=-1*) slope as a target, but changing the identity of the selected map (e.g. selection for plasticity is replaced with selection for genetic evolvability). Again, those simulations are stopped once an average population fitness of $\dot{W}=0.9$ is reached, and the time required for that is recorded. Then, pairwise comparisons (Wilcoxon Signed-rank Test) are made between the average number of generations required to reach a $\dot{W}=0.9$ under the "null" scenario with no previous evolutionary history (dark grey boxplots) and under this later scenario of previous selective events (coloured boxplots). Such a comparison reveals that the evolution of a map takes longer if (*any* of) the maps had evolved before to match a different target (since evolution has to "undo" the already evolved biases before evolving new ones). Whiskers represent extreme values, and boxplot borders the upper and lower quartiles (middle line in the box denotes arithmetic mean). A significance level of *** denotes a $p\text{-}value<10^{-4}$; ** → $p<10^{-3}$ and * → $p<10^{-2}$. $n=30$ replicates. GRN + multilinear model.

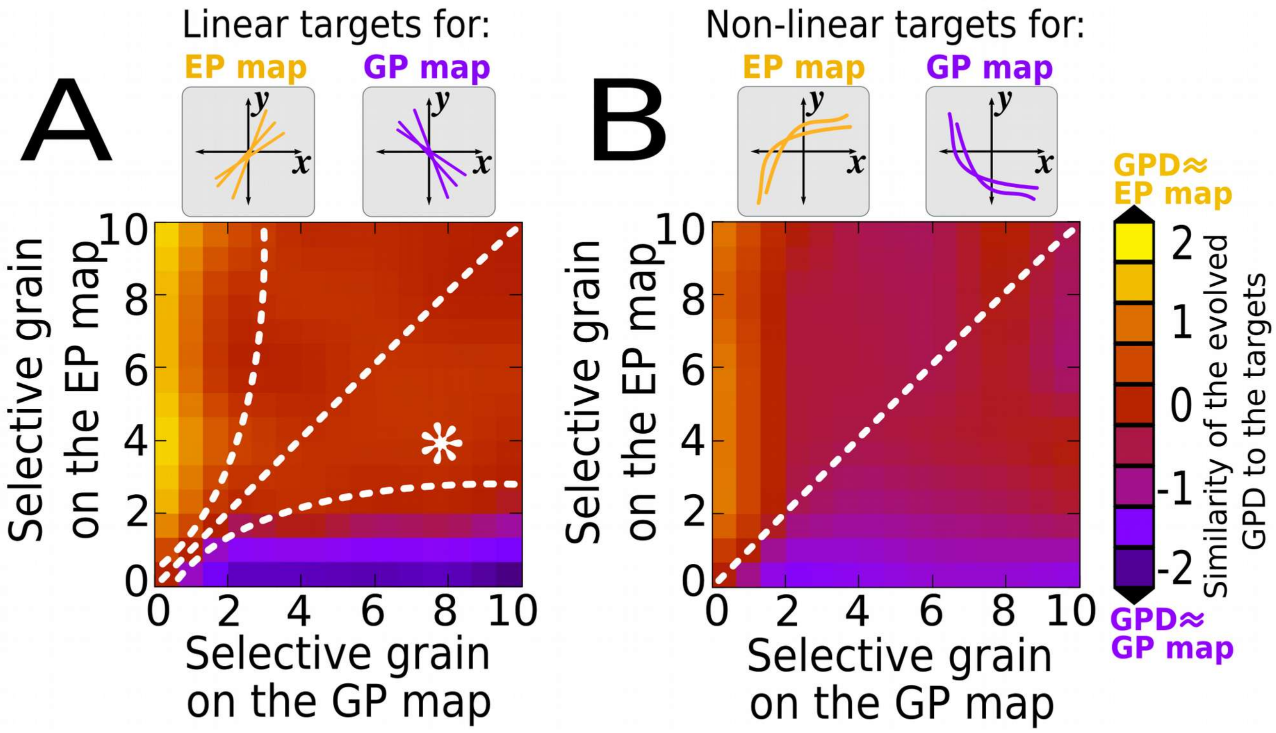

Fig. S8. The structure of the GPD is determined by the map under more fine-grained selection. For this experiment, two different maps (EP map and GP map), each one having a different selective grain, are simultaneously evolved. (A) In order to discriminate between the effects of selection on the EP map and on the GP map, the slopes of the target maps are maximally different: $S^T_{EPM}=(S^T_{GPM})^{-1}$. Then, we vary the selection fine-grainedness (i.e. how many "points" in the evolving maps are selectable) in each of the two selected maps. For instance, the white asterisk represents a population in which the fitness is calculated taking into account four points of the EPM and eight points of the GPM (in both cases, in each generation these points are randomly selected from a set of 10). Colours represent the ED-based similarity of the general phenotypic distribution (GPD) to the targets after t=10^4 generations $ED(GPD,GPM_T)-ED(GPD,EPM_T)$. Yellow colour indicates that trait covariation found in the GPD closely resembles that of the S^T_{EPM}, and blue colour that of the S^T_{GPM}. The resulting heatmap shows that the structure of the GPD is determined by the map under more fine-grained selection. The pattern is most clear when selection on one of the maps is very fine-grained (off-diagonal dashed lines) and in the other map is very coarse-grained. When the selective grain of the two maps is comparable, or when the selection on a map is not very fine-grained, intermediate maps not attributable to any target are found. (B) As in (A) but using non-linear targets (a random polynomial function with $deg(f)>2$). As Fig. 5 shows, the ability of the developmental systems to evolve non-linear maps is severely limited, so here most of the evolved maps are different from both targets (if the target map is too non-linear, nor the genotype nor the environment can be the leaders of adaptive change). $n=30$ replicates, $p=64$ individuals, GRN + multilinear model.

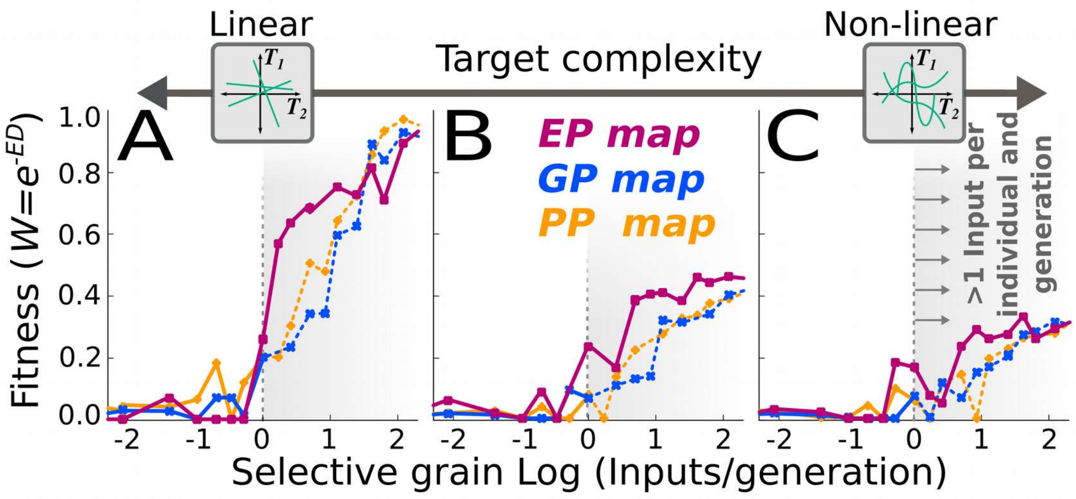

Fig. S9. Simpler maps evolve more easily than complex ones. As in Fig. 5, this figure shows the efficiency of natural selection in evolving certain maps (fitness achieved, vertical axis) under different regimes of selective grain (the average number of parameter-phenotype points that can be "seen" by natural selection in each generation, horizontal axis). In this experiment, however, we use also different non-linear functions (random polynomials) as targets maps. (A) shows the same result shown in Fig. 5: Linear maps can be easily evolved given certain degree of selective grain. (B-C) generalise this result to non-linear target functions: complex maps are hard to evolve even under fine-grained selection regimes. This suggests that the adaptive evolution of very complex maps would require biologically unrealistic levels of selective grain (although they could be attained by means of non-adaptive mechanisms, see main text). For a given target complexity, in particular for simpler ones, maximal efficiency is generally achieved when single individuals can experience more than one input per generation (*Log(Input/Generation)>0*, shadowy areas). Biologically, these high levels of selection fine-grainedness can only be achieved by the Environment-to-Phenotype (EP) map (see main text and Fig. 5). For the sake of clarity, only averages over the $n=30$ replicates are shown (points), and Log-scale is used in the horizontal axis. Euclidean-distance (*ED*)-based fitness. $p=64$ individuals; $t=10^4$ generations, GRN + Multilinear model. For each replicate, the target map is a polynomial function of known degree (complexity) and arbitrary non-zero coefficients.

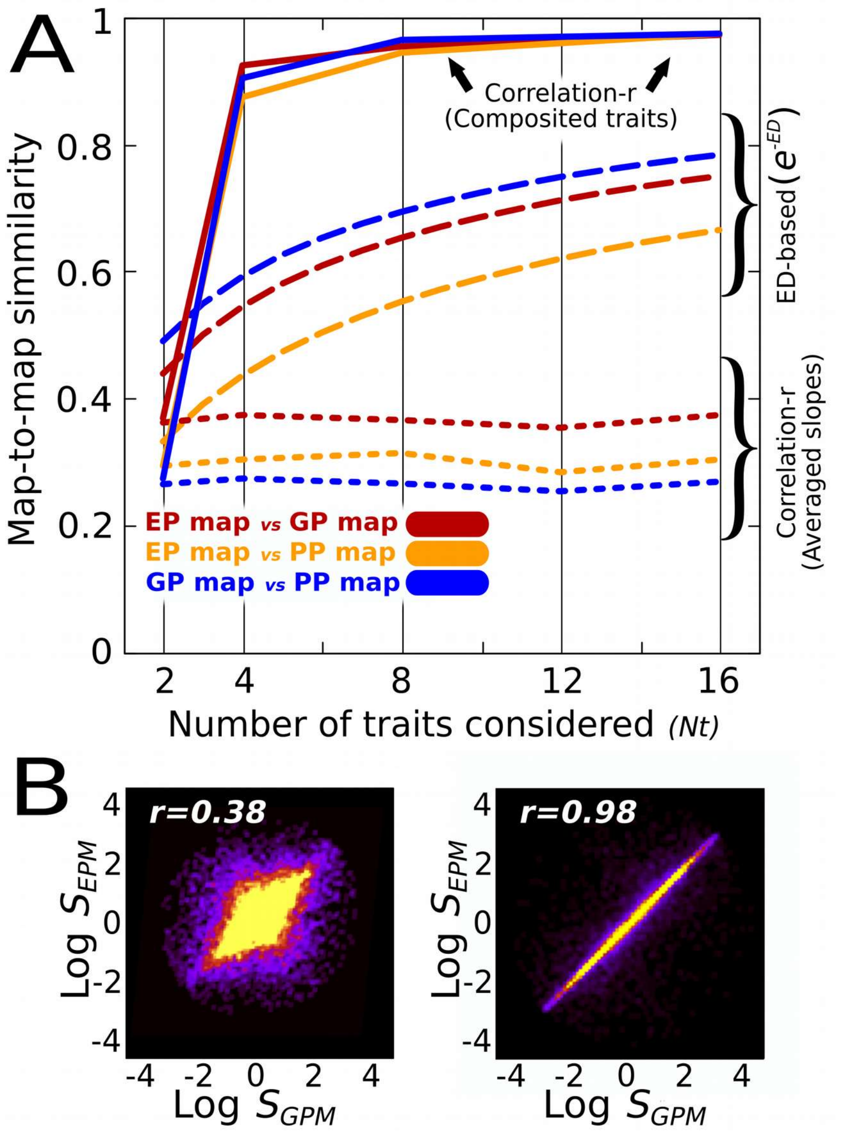

Fig. S10. Taking into account a larger number of traits does not compromise the map-to-map correlations found in previous experiments. (A). The possible effect of the number of traits considered (*Nt>2*) on the map-to-map comparisons was assessed using three independent methods: Composited traits (solid lines), averaged slopes (dashed lines) and Euclidean-based comparison (dotted lines) (see Methods sections, Eqs. 7 and 8). The results suggest that increasing the number of traits does not compromise the map-to-map correlations described throughout this work. Indeed, under the two latter methods the map-to-map similarity does increase as the number of considered traits increases, which is probably explained by the higher degree of pleiotropy the GRN is forced to acquire (in these simulations N_g is kept constant at $N_g=24$, so that as *Nt* increases the average number of character controlled by each of the underlying genes must increase). Contrarily, under the averaged slopes method the correlations remain unaffected by *Nt*, which suggests that our analysis are insensitive to specific choices on the focal traits (e.g. correlations of T3-T4 should give, on average, the same slope than T1-T2, or in general, $S_{1,2} \approx S_{x,y}$, $\forall$ $x \neq y$; $x,y \leq Nt$). (B) Representative two-slope spaces showing, respectively, the moderate and high correlations found for *Nt=2* and *Nt=16* under the composited traits method (each dot is a GRN from the same large ($n=10^6$) random ensemble used for previous experiments (e.g. Fig. 2), and the yellow shaded region contains 90% of the GRNs). *n=30* replicates, GRN + multilinear model, $N_g=24$, *Devt=24.*
